# A basal ursine bear (*Protarctos abstrusus*) from the Pliocene High Arctic reveals Eurasian affinities and a diet rich in fermentable sugars

**DOI:** 10.1038/s41598-017-17657-8

**Published:** 2017-12-18

**Authors:** Xiaoming Wang, Natalia Rybczynski, C. Richard Harington, Stuart C. White, Richard H. Tedford

**Affiliations:** 1 Department of Vertebrate Paleontology, Natural History Museum of Los Angeles County, 900 Exposition Blvd, Los Angeles, CA 90007 United States; 2 Institute of Vertebrate Paleontology and Paleoanthropology, Chinese Academy of Sciences, Beijing, 100044 China; 3 Division of Paleontology, American Museum of Natural History, Central Park West at 79th Street, New York, New York 10024 United States; 4 Palaeobiology, Canadian Museum of Nature, PO Box 3443 STN “D”, Ottawa, Ontario K1P 6P4 Canada; 5 Department of Biology & Department of Earth Sciences, Carleton University, 1125 Colonel By Dr, Ottawa, ON K1S 5B6 Canada; 6 School of Dentistry, University of California, Los Angeles, 10833 Le Conte Ave., Los Angeles, California 90095 United States

## Abstract

The skeletal remains of a small bear (*Protarctos abstrusus*) were collected at the Beaver Pond fossil site in the High Arctic (Ellesmere I., Nunavut). This mid-Pliocene deposit has also yielded 12 other mammals and the remains of a boreal-forest community. Phylogenetic analysis reveals this bear to be basal to modern bears. It appears to represent an immigration event from Asia, leaving no living North American descendants. The dentition shows only modest specialization for herbivory, consistent with its basal position within Ursinae. However, the appearance of dental caries suggest a diet high in fermentable-carbohydrates. Fossil plants remains, including diverse berries, suggests that, like modern northern black bears, *P*. *abstrusus* may have exploited a high-sugar diet in the fall to promote fat accumulation and facilitate hibernation. A tendency toward a sugar-rich diet appears to have arisen early in Ursinae, and may have played a role in allowing ursine lineages to occupy cold habitats.

## Introduction

**Figure 1:**
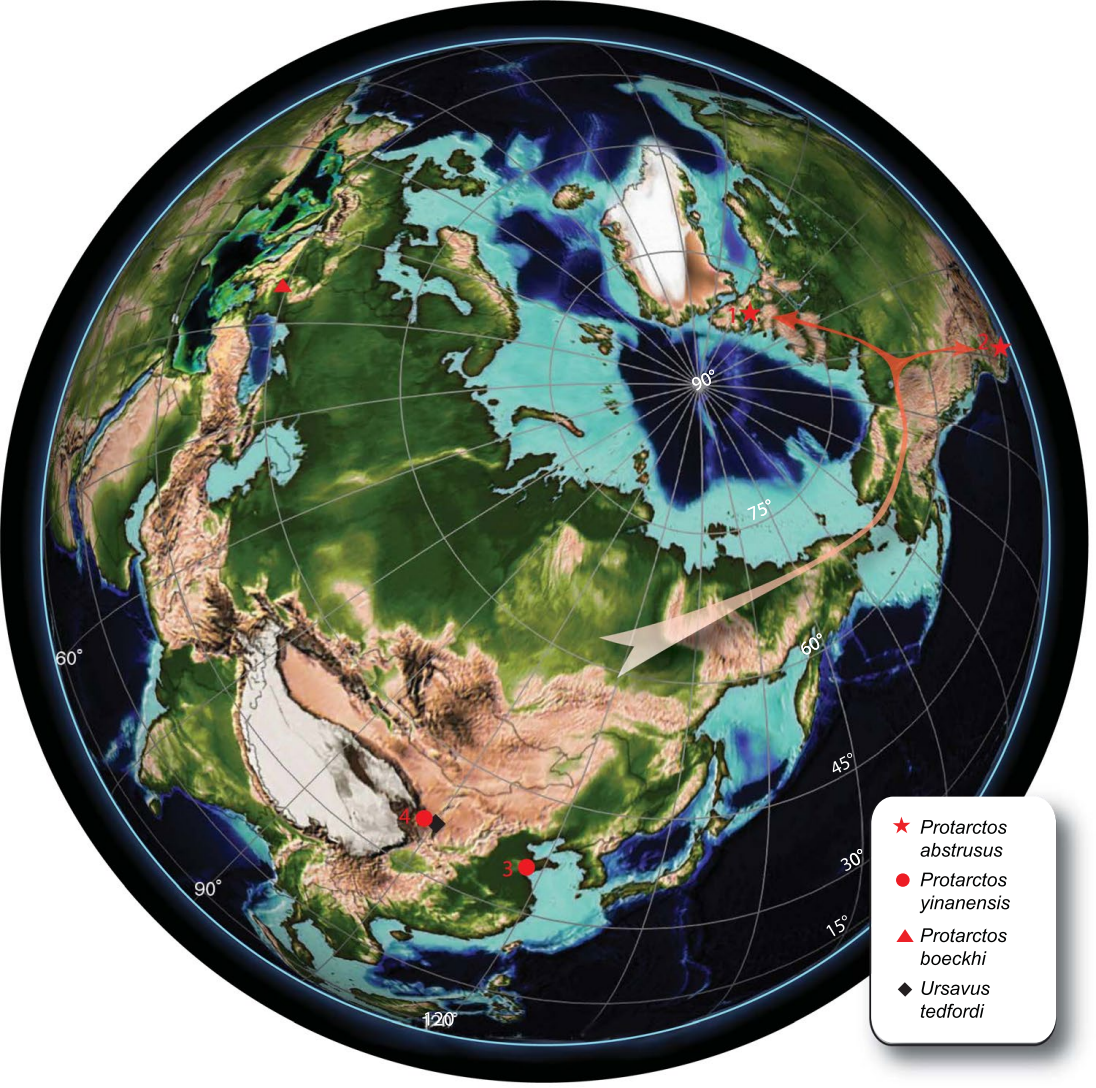
Map of key basal ursine localities in Asia, Europe, and North America and routes of dispersal. The Beaver Pond site is indicated by red star 1 within Arctic Circle and the type locality of *Protarctos abstrusus* in Idaho is the red star 2, near the edge of the map. For much of the Neogene the Bering isthmus would have served as a land bridge, allowing for an Arctic biotic continuity between Eurasia and North America. By 3.5 Ma the Bering Strait was open, although mammalian dispersal could have been permitted by seasonal sea ice. Pliocene (5 Ma) paleogeography map modified from Wang *et al*.^86^ Fig. 1 and Scotese^87^.

In 1970, Philip Bjork described a small fossil bear from the Pliocene Glenn’s Ferry Formation of southwestern Idaho. Based on a single m1 as the holotype, he was understandably perplexed and named it *Ursus abstrusus*. Additional material has not been forthcoming since its initial description and this bear has remained an enigma. Hence the discovery in the 1990s of a similar bear from more complete fossils in the Pliocene of the Canadian High Arctic throws much needed light onto the mystery (Fig. 1). In addition to resolving the riddle of *Ursus abstrusus*, with a moderately complete skull and lower jaws with associated postcranials, the new materials present a rare opportunity to fill a large gap in our knowledge of North American High Arctic at a time in the early Pliocene when mean annual temperatures in the High Arctic were ~22 °C warmer than the present polar temperatures^1^. Such a warm climate supported an extensive boreal-type forest biome^2,3^, radically different from today’s arid polar tundra^4^. Thus the evidence of this primitive bear in an extinct polar forest offers valuable information about the diet and habitat of this basal ursine.

The fossil records of basal ursines has improved with recent discoveries of three relatively complete specimens of basal ursines from China – a very advanced *Ursavus* ^5^ and a very primitive *Protarctos* ^6,7^. We are now in a position to more tightly bracket the North American Pliocene bears as well as providing a wealth of information about cranial anatomy of basal ursines previously unavailable. The present description of *P*. *abstrusus* and a phylogenetic analysis combining molecular and morphological data of most fossil and living ursines for the first time allows a much more detailed view of the history of bears at the critical juncture of their initial diversification. In addition, the presence of dental caries provides insight into the evolutionary history of diet of ursines.

## Systematic Paleontology

Order Carnivora Bowdich,1821^8^.

Family Ursidae Fischer van Waldheim, 1817^9^.

Subfamily Ursinae Fischer von Waldheim, 1817.

Tribe Ursini Fischer von Waldheim, 1817.

Protarctos Kretzoi, 1945^10^.

## Genotypic Species

*Protarctos boeckhi* ^11^.

## Included Species

*Protarctos boeckhi* ^11^; *P. abstrusus* (Bjork, 1970); *P. yinanensis* (Li, 1993); and *P. ruscinensis* ^12^.

## Distribution

Pliocene of Europe, Pliocene and early Pleistocene of Asia, and Pliocene of North America.

## Emended diagnosis

**Figure 2:**
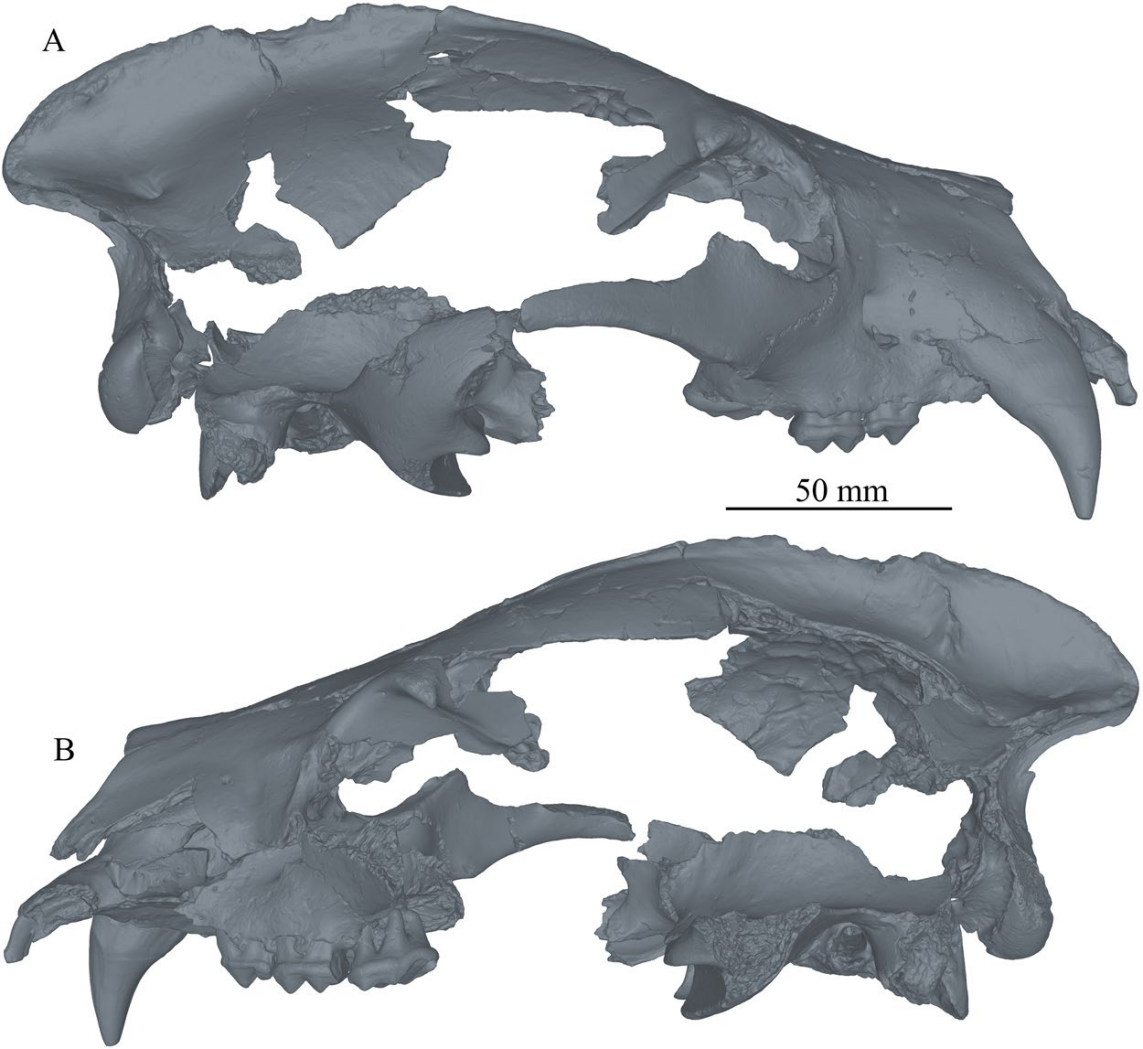
Right (**A**) and left (**B**) lateral views of the skull of *Protarctos abstrusus* (CMN 54380), composite laser scans of five individual cranial fragments, assembled in Avizo Lite (version 9.0.0) and visualized in PointStream 3D Image Suite (Version 3.2.0.0).

**Figure 3:**
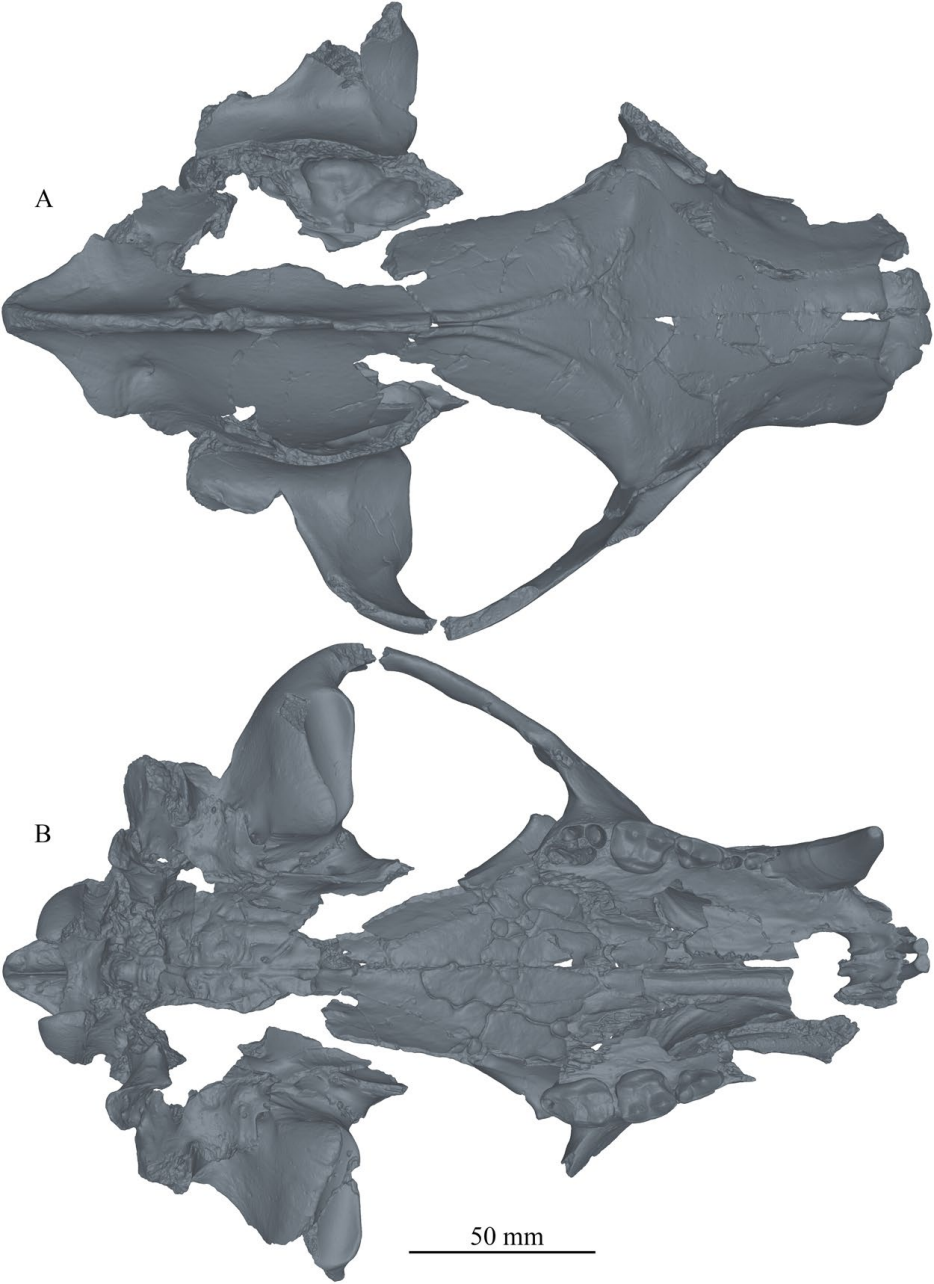
Dorsal (**A**) and ventral (**B**) views of the skull of *Protarctos abstrusus* (CMN 54380), composite laser scans of five individual cranial fragments, assembled in Avizo Lite (version 9.0.0) and visualized in PointStream 3D Image Suite (Version 3.2.0.0).

**Figure 4:**
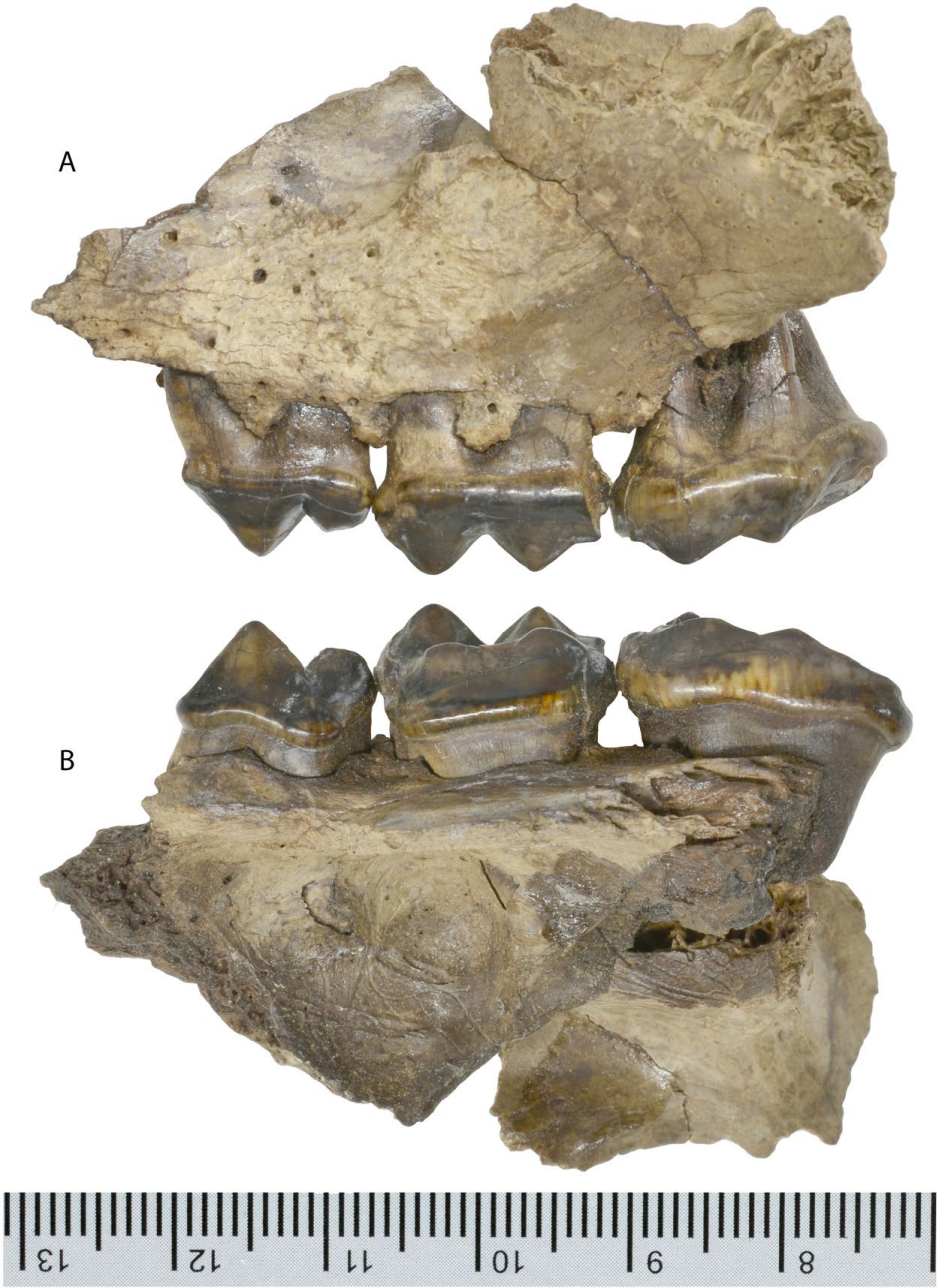
Left upper posterior teeth (P4-M2) of *Protarctos abstrusus*, CMN 54380; (**A**) buccal, and (**B**), lingual views.

**Figure 5:**
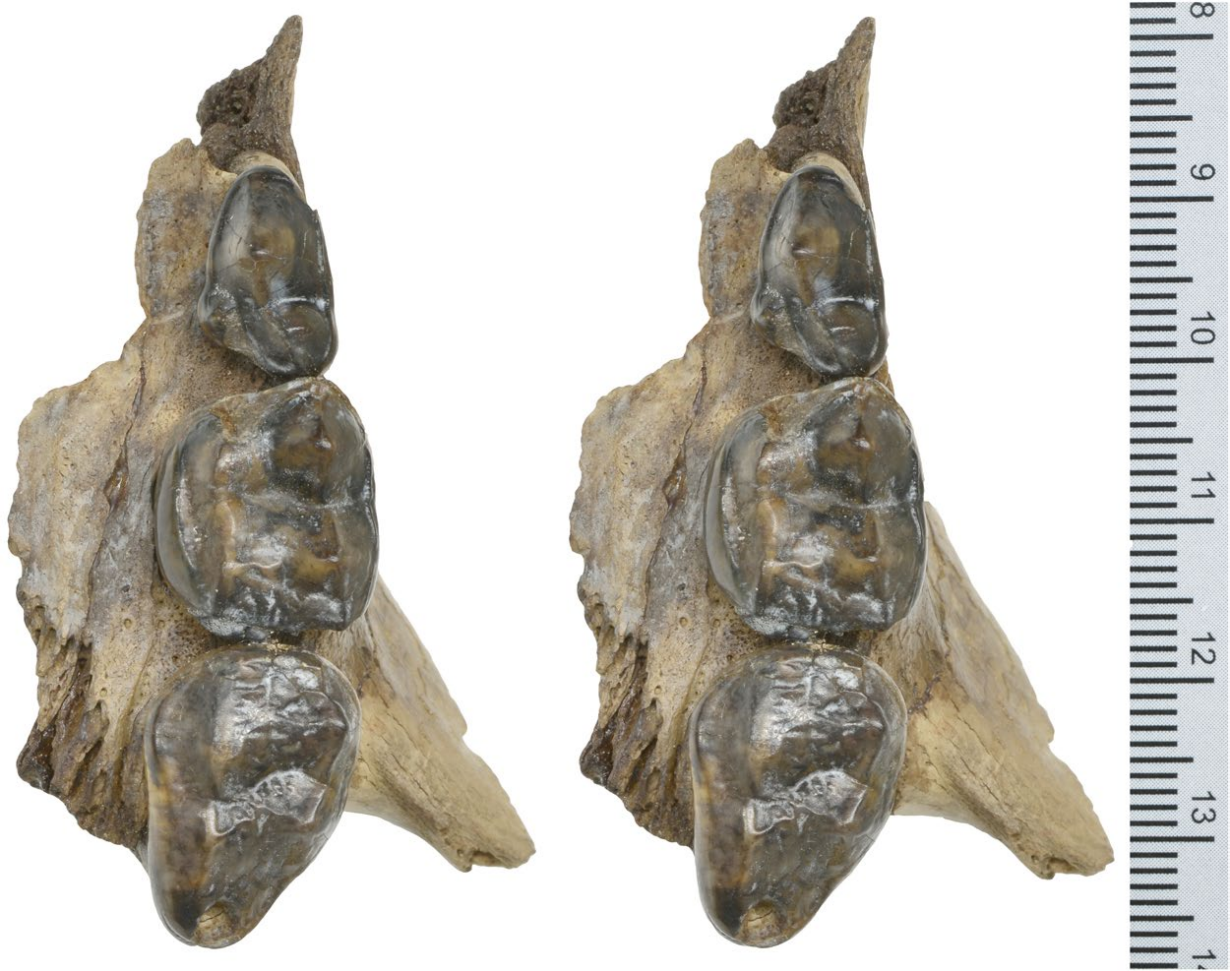
Stereo photos of left upper posterior teeth (P4-M2) of *Protarctos abstrusus*, CMN 54380.

**Figure 6:**
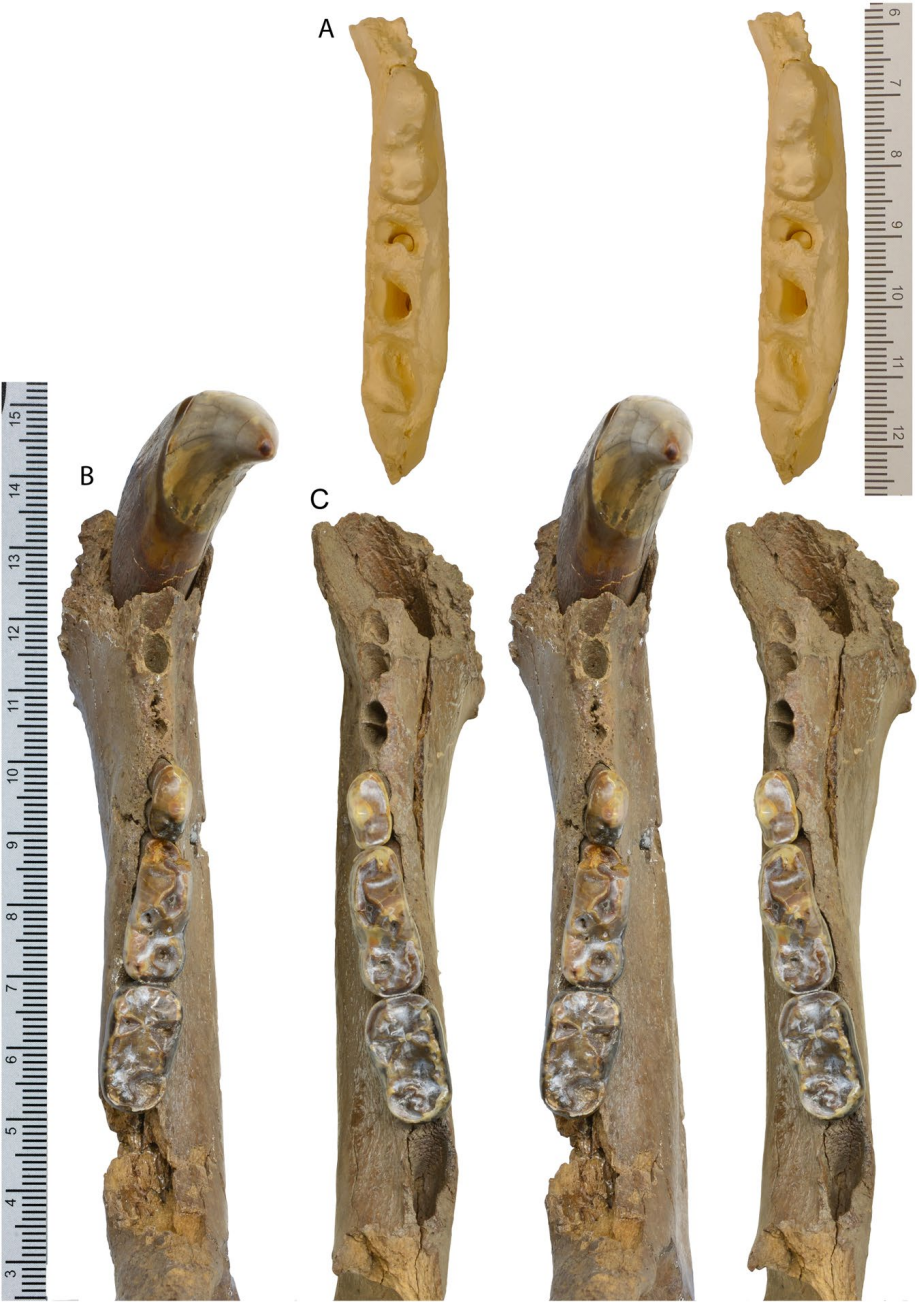
Stereo photos of lower cheek teeth of *Protarctos abstrusus*, occlusal views. (**A**), holotype, a cast of UMMP V53419 (cast, USNM 170872) from the Hagerman fossil site in Idaho, (**B**), right (CMN 52078B), and (**C**), left (CMN 52078A) dentaries from the Beaver Pond site in Nunavut.

*Protarctos abstrusus* is a basal ursine the size of a small Asian black bear. It has a flat forehead covering an uninflated frontal sinus; very high sagittal crest that projects backward to overhang the occipital condyle (Figs 2 and 3); P4 with a small, distinct protocone situated at the level of carnassial notch; M2 talon modestly developed but not very elongated (Figs 4 and 5); no pre-metaconid on m1, smooth posterior surface of m1 trigonid without zigzag pattern, presence of a distinct pre-entoconid; m2 shorter than m1 (Fig. 6 and Supplementary Fig. S2). It is about the same size as *P*. *boeckhi* and differs from it in the relatively smaller p4, presence of a tiny cuspule on lingual side of posterior crest in p4, and presence of a pre-entoconid on m1. *P*. *abstrusus* is also similar in size to *P*. *yinanensis* and can be distinguished from the latter in a flattened forehead, posteriorly projected sagittal crest, p4 posterior accessory cuspule on lingual side of posterior crest, an m1 pre-entoconid, and less elongated M1 and M2. *P*. *abstrusus* differs from *P*. *ruscinensis* by its lack of unique features of the latter such as a deep angular process, reduction of P4 protocone, and a single entoconid on m1.

## Taxonomic Remarks

There is much disagreement over the generic taxonomy of ursines. Most mammalogists and some paleontologists include all living black bears (Asian and North American), brown bears, and polar bears in the genus *Ursus* but allow separate generic status for the sloth bear, *Melursus*, and sun bear, *Helarctos* ^14–19^, although some include all of above in *Ursus* ^20,21^ and others use *Thalarctos* for the polar bear^22,23^. With a deep time perspective, vertebrate paleontologists either adopt some subgeneric names, such as *Ursus* (*Melursus*) for sloth bear, *Ursus* (*Selenarctos*) for Asian black bear, *Ursus* (*Euarctos*) for American black bear, *Ursus* (*Protarctos*) for some extinct bears^7,24–26^ or elevate some of them to generic status^6,27^. In his remarks about carnivoran classification, Kretzoi^10^ erected a new genus, *Protarctos*, for *Ursus boeckhi* Schlosser, 1899. Kretzoi’s name has been adopted either at full generic rank^6^ or as a subgenus^7^, although many authors still prefer a more inclusive usage of *Ursus* ^19,24,25,28–30^. In our cladistic framework in this study, some generic reassignment becomes necessary to maintain monophyly, especially in light of the general preference to giving sloth and sun bears distinct generic status.

*Protarctos abstrusus* (Bjork, 1970), new combination.

*Ursus abstrusus* Bjork, 1970: Ruez 2009:43.

## Holotype

UMMP V53419 (locality UM-Ida 79-65), left dentary fragment with p4 alveolus, m1, and m2-3 alveoli (Fig. 6 and Supplementary Fig. S6) from Glenn’s Ferry Formation, southwestern Idaho; Hagerman Local Fauna, 3.48–3.75 Ma, early Pliocene.

## Referred Specimens

CMN 54380 (accession number CR-97-18; same as below), a fragmentary partial skull including much of dorsal roof, left and right maxillary, partial left and right basicranial area, and isolated left and right petrosals, with left I1, I2-3 alveoli, P4-M2, and right I1, I2-3 alveoli, C1, P1-3 alveoli, P4-M1, and M2 alveolus; CMN 52078-A (CR-93-8A), partial left dentary with detached canine (CR-92-24), p1-3 alveoli, p4-m2, and m3 alveolus; CMN 52078-B (CR-93-8B), partial right dentary with detached canine, p1-3 alveoli, p4-m3; CMN 51779-A (CR-97-33A), nearly complete left and right pelvis; CMN 51779-B (CR-97-33B), nearly complete left femur; CMN 53990 (CR-92-1), nearly complete axis vertebra; NUFV 303 (SF-06-15), nearly complete right radius; NUFV 304 (SF-06-17), cervical vertebrae, C3; CMN 53989 (CR-92-2), partial lumbar vertebra (museum label: 7th?); CMN 53984 (CR-95?-0), left tarsal IV; CMN 53988 (CR-96-43), right metacarpal III (museum label: slightly smaller than a modern male black bear; maximum length 68.4); CMN 53982 (CR-93-69), metacarpal or metatarsal IV (maximum length 60.2); CMN 53985 (CR-95-33), proximal phalanx (maximum length 33.0 mm); CMN 53980 (CR-93-36), proximal phalanx (maximum length 36.5); CMN 53981 (CR-93-43), left medial phalanx (maximum length 28.7 mm); CMN 53983 (CR-94-102), medial phalanx (maximum length 22.6 mm; width 14.5 mm); CMN 53987 (CR-96-31), distal phalanx (maximum length 31.3 mm).

## Locality and Age

The Beaver Pond site, 78° 33′N 82° 22′W, is a >20 m succession of fine to coarse cross-bedded fluvial sands conformably overlain by cobble gravels interpreted to be glacial outwash and capped by 2 m of till on the northeastern edge of an interfluvial plateau southeast of Strathcona Fiord on Ellesmere Island, Nunavut^31,32^ (red star 1 in Fig. 1). A peat deposit near the base of the sequence, up to 2.4 m thick, produced exceptionally well-preserved plant, invertebrate and vertebrate remains (Supplementary Fig. S2), and is disconformably overlaying light-colored, tilted Eocene sediments. Abundant beaver-cut branches and cut saplings of larch trees suggest that the peat growth may have been promoted by beaver activity. Further supporting this view are the skeletal remains of multiple beaver individuals, and two clusters of beaver-cut branches found within the peat unit, at least one of which was interpreted to be the core of a dam^32,33^. Using terrestrial cosmogenic nuclide (TCN) burial dating^34^, four samples of quartz-rich coarse sand from above the peat unit yielded a weighted mean date of >3.4 + 0.6/−0.4 Ma, suggesting the peat accumulation was formed during a mid-Pliocene warm phase^31^.

## Paleoenvironments and Associated Flora and Fauna

At 78°N, the Beaver Pond site on Ellesmere Island is presently extremely cold and arid, with ice sheets, permafrost, and sparse vegetation. During the mid-Pliocene, the Canadian High Arctic would have been forested, and the latitudinal gradient was much less than modern, so that although global temperatures were 3-4 degrees warmer than modern, the mean annual temperature of the terrestrial High Arctic was ~22 °C warmer (Fletcher *et al*. 2017).

The Beaver Pond site comprises the remains of a Pliocene forest wetland community that was dominated by larch (*Larix groenlandii*), and also supported alder (*Alnus*) and birch (*Betula*), spruce (*Picea*), pine (*Pinus*) and cedar (*Thuja*)^1^. Multiple proxies consistently suggest a Pliocene mean annual temperature at the Beaver Pond site of slightly above freezing, with plant community composition indicating a warmest summer air-temperatures of ~20 °C^1^. Coldest winter temperatures have been recently estimated from vegetation to be ~−12 °C, though a prior estimation from beetle fauna suggest −27 °C^1^. Precipitation at the Beaver Pond site was also much greater in the Pliocene. Modern (1960–1990) Mean Annual precipitation in the area is 104 mm/year, whereas in the Pliocene the plant community implies precipitation to have been ~550 mm/year^1^.

In fossil vertebrates, the Beaver Pond site, in combination with the nearby Fyles Leaf Bed fossil site, has produced four native North American mammals: a castoroidine beaver *Dipoides* sp., an archaeolagine rabbit *Hypolagus* cf. *H*. *vetus*, a small canine dog *Eucyon*, and a cameline camel (c.f. *Paracamelus)* ^31,35^. Of these, *Eucyon* and *Paracamelus* had arrived at Eurasia near the Mio-Pliocene boundary, and they may be closely related to the ancestral stock that gave rise to the Eurasian forms. The rest of the faunal components include a frog, a percid fish, *Sander teneri*, of Eurasian origin, and ten mammal taxa which share considerable similarity to equivalent-aged faunal assemblages in East Asia, including a neomyine shrew *Arctisorex polaris*, a microtine-like cricetid similar to *Microtodon* or *Promimomys*, a large wolverine (cf. *Plesiogulo*), a fisher (*Martes/Pekania*)-like carnivore, a marten-like carnivore *Martes* cf. *M*. *americana*, a weasel *Mustela* sp., a meline badger *Arctomeles sotnikovae*, a three-toed horse *Plesiohipparion*, a possible cervoid *Boreameryx braskerudi* of unknown origin, plus an ursine bear “*Ursus abstrusus*” described herein^4,32,35^. The third author has also identified a duck closest to the Greater Scaup (*Aythya marila*).

## Distribution

Known only in the Pliocene (Blancan) of southwestern Idaho and Ellesmere Island, Nunavut of Canadian Arctic. A possible record from Buckeye Creek Local Fauna of Nevada has been attributed to this species^36^, but it is too poorly known to be certain.

## Description

Skeletal remains of the fossil bear were collected in different years (1992, 1993, 1996, 1997, 2006) from the Beaver Pond site (Supplementary Fig. S1). The skull specimen, with upper teeth (CMN 54380) appears to be a young adult (Figs 2–5, Supplementary Part 3 and Figs S3–5). The exoccipital-basioccipital, exoccipital-supraoccipital, and premaxilla-maxilla sutures are largely fused, whereas the internasal and interfrontal elements are unfused. In the modern black bear this degree of fusion of the cranial elements suggests the individual is between five and seven years old^13^. The upper teeth, particularly the premolar and molar cheek teeth, are essentially pristine and show wear only on the tip of the upper right canine, incisors, and anterior edge of M1, which also suggest a relatively young individual (Figs 4 and 5). In contrast, there is extensive wear on the lower teeth (CMN 52078-A and CMN 52078-B), indicating the mandibles are from an individual much older than that of the cranium (Fig. 6 and Supplementary Fig. S6). The symphysial sutures of the left and right dentaries occlude perfectly, and the wear patterns on the lower teeth on either side are comparable, indicating a single individual for the lower jaws. There are thus a minimum of two individuals. Judging by the lack of fusion between epiphysis and diaphysis, the postcranial elements may belong to the younger individual represented by the skull (CMN 54380).

## Results

### Phylogenetic analysis

**Figure 7:**
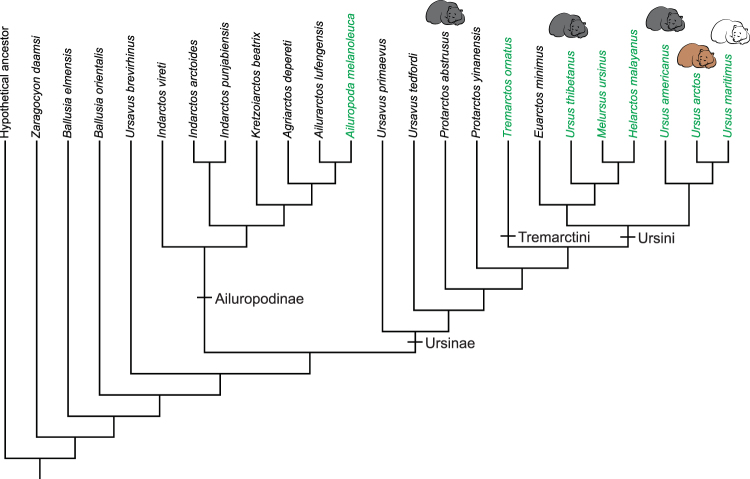
Cladogram of select extinct and extant ursids based on our character matrix (Supplementary Table S6) within a molecular backbone phylogeny of Kutschera *et al*.^37^ Fig. 2A. This tree is six steps longer than the unconstrained tree (see text for explanation). Taxa in green represent living bears. Sleeping bear symbols indicate hibernators.

A phylogenetic analysis was conducted using 24 taxa and 59 morphological characters (Supplementary Tables 5–6. The taxa included five fossil ursines (*Ursavus primaevus*, *U*. *tedfordi*, *Protarctos abstrusus*, *P*. *yinanensis* and *Euarctos minimus*) and all seven living ursines (*Tremarctos ornatus*, *Melursus malayanus*, *Ursus thibetanus*, *U*. *americanus*, *U*. *arctos* and *U*. *maritimus*). A new single shortest tree was found by New Technology search in TnT with a tree length of 145, consistency index of 0.51, and retention index of 0.70 (Fig. 7). The topology of modern taxa was constrained using nuclear DNA evidence of Kutschera *et al*.^37^ and the whole genome analysis of Kumar *et al*.^38^. Assuming the molecular relationship is correct, six extra morphological steps (homoplasies) are required to account for this new relationship. *Protarctos abstrusus* appears basal to all modern bears, including *Tremarctos*, the spectacle bear of South America. Moreover, its phylogenetic position suggests a Eurasian origin for this lineage. Asia appears to be of vital importance in the early diversification of ursines: Not only is Asia home to all basal ursines still alive today (sloth bear, sun bear, and Asian black bear) but the most advanced stem form, *Ursavus tedfordi*, leading to the ursines is also found in east Asia^5^, as well as early ursines such as *Ursus yinanensis* ^6,7^. (see SI for further discussion).

### Body mass estimate

Using regression parameters derived from species of living Ursidae, log_10_ (body mass) = 2.02*log_10_ (skull length) −2.80^39^. Table 10.2, we arrive at an estimated body mass of 97 kg for *Protarctos abstrusus* from its skull length (condylobasal length of Table S1) of 234 mm (CMN 54380). If m1 length is used (20.1 mm, see Table S2), a less desirable proxy^39^, an estimate of 79 kg results. In absence of more superior proxies such as long bone cortical thickness^40,41^, body mass estimate based on skull length is preferred here. *P*. *abstrusus* is thus close to average male body mass of American black bear from California (86 kg) and heavier than their female counterparts (58 kg)^42^.

### Dental Caries

**Figure 8:**
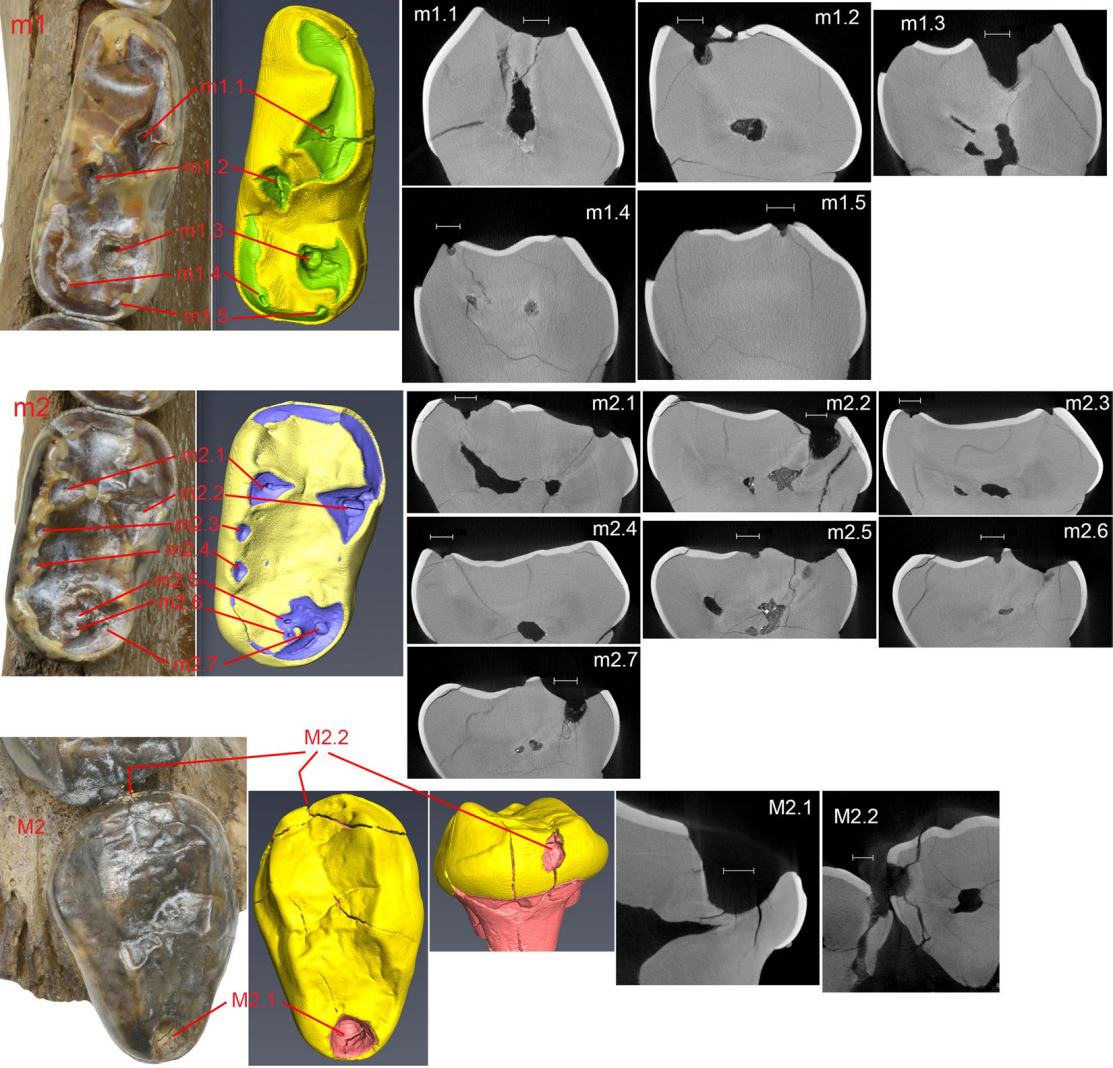
MicroCT scans of *U*. *abstrusus* dental caries; all cross sectional images oriented buccolingually through depth of lesion except as noted. **m1** (CMN 52078-A), occlusal view of m1 (reversed from left side) and reconstructed view of m1 with enamel in yellow and dentin in green; **m1**.**1**, worn and slightly elevated surface with no caries (as control); **m1**.**2**, small dentinal caries; **m1**.**3**, note demineralization of dentin wall extending approximately 0.1 mm and dentinal sclerosis pulpal to depth of lesion; **m1**.**4**, small carious lesion, note also loss of enamel on lingual surface due to breakage; **m1**.**5**, small carious lesion. **m2** (CMN 52078-B), occlusal view of m2 and reconstructed view of m2 with enamel in yellow and dentin in blue; **m2**.**1**, small lesion on left; **m2**.**2**, carious lesion revealing demineralization of the pulpal surface, demineralization of the dentinal tracts extending to the pulp, and probably reparative (secondary) dentin formation in the pulp underlying the demineralized dentinal tracts.; **m2**.**3**, early lesion on left; **m2**.**4**, early lesion; **m2**.**5**, early lesion; **m2**.**6**, small lesion; **m2**.**7**, note areas of demineralization on the periphery of the lesion and demineralization and deeper sclerosis of dentinal tubules running towards the pulp. **M2** (CMN 54380, left side), occlusal view of M2 and reconstructed view of M2 with enamel in yellow and dentin in pink; M2.1, note subsurface demineralization at depth of lesion, deeper sclerosis of dentinal tubules, and apparent constriction of distal pulp horn by reparative dentin; M2.2, slice oriented mesiodistally through depth of lesion, note subsurface demineralization in depth of this proximal surface lesion, deeper sclerosis of dentinal tubules, and mild constriction of pulp horn by reparative dentin. Note also proximal surface caries of distal surface of adjacent M1. Scale = 1.00 mm.

Judging from dental wear, the partial skull (CMN 54380) and mandibles (CMN 52078-A, B) from the Beaver Pond site represent two individuals of *Protarctos abstrusus*. Both show evidence of dental caries, particularly on teeth that have sustained the most wear; a pit usually develops on the exposed dentine surface (Fig. 8). We used microCT scanning to investigate features of the left upper second left molar (M2), and the right side lower first (m1) and second molars (m2) of the mandibular specimen, CMN 52078-A. The M2 has deep occlusal (Fig. 8, M2.1) and proximal (Fig. 8, M2.2) surface lesions. Scans show that both lesions are characterized by a thin zone of demineralization at the cavity boundary and deeper sclerosis of the dentinal tubules. There is also evidence of mild formation of reparative (secondary) dentin formation in the adjacent pulp associated with each lesion. The lower carnassial, m1, revealed five structures of interest (Fig. 8, m1.1–m1.5). Feature m1.1 (Fig. 8, m1.1) is a fragment of dentin that is slightly elevated from a worn surface because of cracks in the desiccated dentin. Feature m1.2 represents a series of carious lesions extending apically to the worn surface (Fig. 8, m1.2). Three small pit-like lesions and one large lesion (feature m1.3) are identified. MicroCT scans reveal that lesions undercut the worn surface and show slight demineralization of their margins. Demineralization of dentinal tubules is a reaction to actively spreading caries while dentinal sclerosis and formation of reparative dentin are evidence of protective responses. Feature m1.3 (Fig. 8, m1.3) demonstrates subsurface demineralization extending about 0.1 mm from the margins of the cavity. Features m1.4 and m1.5 are early carious lesions (Fig. 8, m1.4 and m1.5, respectively) that clearly extend below the worn surface. Seven features of interest were identified in the occlusal surface of m2 (Fig. 8, m2.1-m2.7). Five early carious lesions are identified under the scale bars of m2.1, m2.3, m2.4, m2.5 and m2.6. Feature m2.2 shows demineralization of the pulpal surface of the lesion. There is also evidence of demineralization of the dentinal tracts between the depth of the lesion and the pulp as well as a mildly sclerotic peripheral zone. Further, it is most likely that some reparative (secondary) dentin has formed in the region of the pulp adjacent to the demineralized dentinal tracts. Feature 2.7 also shows slight demineralization of the lesion surface as well as slight demineralization of dentinal tracks just pulpal to the lesion as well as deeper sclerotic changes.

For comparative purposes, we assessed the prevalence of caries in modern American black bear populations (*Ursus americanus*) using museum collections, as well as published data from museum collections and a living population. Dental caries in extant black bears are seen in both museum specimens and *in vivo* bears (0–44% prevalence) (Supplementary Table S3), in contrast to their general absence in other carnivores^43^. Moreover, examination of northern boreal forest black bears from Canadian Museum of Nature collections revealed prevalence of caries increasing with age (Supplementary Table S4).

## Discussion

**Figure 9:**
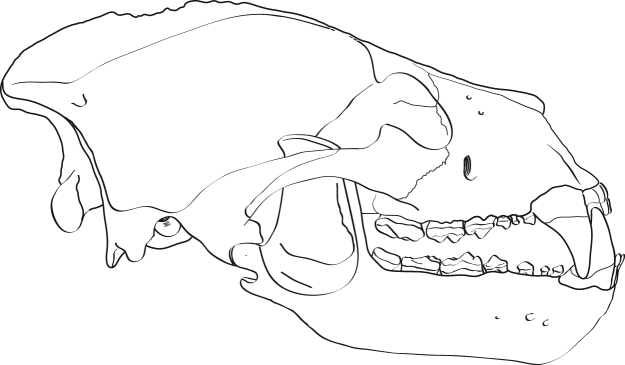
Artist restoration of lateral view of skull and lower jaw of *Protarctos abstrusus* based on a composite of partial skull (CMN 54380) and right dentary (CMN 52078-B). Missing bones (ascending ramus, mandibular condyle, and angular process) are based on living black bears. Missing teeth (I2-3, P1-3, i2-3, p1-3, and m3) are restored based on their alveoli. The stage of wear on the lower teeth is drawn to match with those of the upper teeth. Art by Xiaoming Wang.

Analysis of new fossil material of *Protarctos abstrusus* from the North America High Arctic shows that, although ecomorphologically similar to the modern North American black bear (*Ursus americanus*), *P*. *abstrusus* represents a basal ursine. The most prominent cranial features of *P*. *abstrusus* are its relatively short rostrum, flat forehead above the orbit, and high sagittal crest that extends posteriorly and overhangs the occipital condyles (Fig. 9), characters that generally signal primitive status within Ursinae. *P*. *abstrusus* appears to have been an isolated immigration event from Eurasia to North America, separate from *Ursus*, representing a time of Asian-North American high latitude floral and faunal interchange^32^, when the high-latitude forests of Asia and North America were connected across the Beringian isthmus.

The American black bear, by contrast, appears in the North America fossil record in the Early Pleistocene as a result of an independent dispersal event from Eurasia. Fossil records of true American black bear, *Ursus americanus* Pallas, range from Irvingtonian to late Rancholabrean^44,45^. From the Irvingtonian age, Brown^46^ described abundant materials from the Conard Fissure, Arkansas, which he referred to *U*. *americanus*. Gidley^47^ named *Ursus* (*Euarctos*) *vitabilis* from the Cumberland Cave, Maryland, which was later referred to *Euarctos vitabilis* ^48^. By late Rancholabrean black bears appear widespread throughout North America^49^. Several species or subspecies of late Pleistocene black bears have been named, which were sometimes confused with brown bears because of overlap in size and pronounced sexual dimorphism^50,51^, such as *Ursus optimus* from late Pleistocene McKittrick brea deposits of southern California^52^, which was determined by Graham^53^ to be a brown bear. Graham^53^ concluded that only one species, *Ursus americanus*, is valid throughout the Pleistocene with late Pleistocene fossil forms being larger than their living descendants, suggesting continuity of the black bear linage in North America, as was also pointed out earlier by Kurtén^54^.

Within Ursinae, *P*. *abstrusus* represents a stage of dental evolution that is intermediate in its specialization for ingesting plants, and significantly less than modern bears - polar bears being an exception, showing evolutionary reversal toward increased carnivory. The evolutionary history of ursines is generally characterized by a shift in dental specialization from carnivory to increased omnivory, with the posteriormost molars of more recent forms being more elongate, and wrinkled, allowing for more crushing surface (Table S2). Although, morphologically, *P*. *abstrusus* is less specialized than modern bears, the presence of dental caries suggests the diet of this 3.5 million-year-old transitional form already included a significant carbohydrate component.

Dental evidence from the beaver pond site *P*. *abstrusus* appears to be from two individuals, including an apparent young adult, and both show dental caries, suggesting their diets included high amounts of fermentable carbohydrates early in their lives. Simple sugars, such as glucose and fructose, are readily metabolized by many bacteria found in the oral biofilm into various acids. These acids demineralize enamel and dentin and may lead to dental caries^55^. Cariogenicity is highly correlated to the amount^56,57^ and frequency^58,59^ of sugar intakes. The type of sugar consumed and associated dental caries are also found to differ. Despite their high sugar content, raw fruits by themselves are not always implicated for cariogenicity, although high frequency (up to 17 times a day) may induce caries^60^. In humans, there is convincing evidence that free-sugar consumption of more than four times a day or more than 6–10% energy intake will increase incidents of dental caries^57^. Historically in humans, increase in prevalence of dental caries has generally been associated with dietary shifts, linked with a reduction of nomadic lifestyles^61^, the development of agriculture in Neolithic populations, and even more so with industrialization^62^.

In bears, carbohydrate intake may account for the appearance of dental caries (Tables S3 and S4), and may also be related to sedentary behavior, particularly for northern bears which hibernate. Northern black bears hibernate five to seven months, and survive better if they have high fat reserves^63^. In bears, the optimal diet for production of fat reserves appears to be one of high-energy carbohydrates (e.g., fruits) and low in protein. High-latitude berries (such as bearberry) often have a wide, circumpolar distribution and can be found in a variety of northern habitats including forest, woodland, wetland and tundra habitats^64^. Black bears and grizzly bears in boreal forest eat berry fruits in the autumn, but some fruits, such as cranberry and bearberry, frequently remain on the vine over winter and are important to bears coming out of hibernation in the early spring^65–67^. Bearberry (*Arctostaphylos uva-ursi*) fruits are relished and highly important to black bear in Pelly River Valley of Yukon Territory^66^. Berries are found in nearly 80% of bear scats collected during the fall period and consistently represent a large component of black bear diet in Alaska, with blueberries (*Vaccinium uliginosum*) being the most common^65^. However, fruit intake may be mitigated by factors such as fruit abundance and body size. For example, larger bodied bears appear to tend toward carnivory, as they are less efficient than smaller bears at exploiting small fruits^68^. These factors may underlie the high variation observed in caries prevalence seen among populations of modern black bears (Table S3).

Floral macrofossils from the Beaver Pond shows a diversity of berries would have been available to *U*. *abstrusus*, including *Empetrum nigrum* (crowberry), *Vaccinium* sp. (e.g., blueberry, lingonberry), *Rubus idaeus* (raspberry)^69^, and their abundance may have been enhanced following forest fires, which is evident at this site^33^. Therefore berries may have constituted a component of the Beaver Pond bear’s diet, particularly during the peak seasons, and their high sugar and acid contents could have resulted in the observed pronounced dental caries. The bear’s habitat may also have included honeybees, but this is speculative. The genus *Apis* includes honeybees that are today the basis of the honey industry. The genus appears to have originated in Europe dispersing into Asia, Africa as well as North America^70^. In North America the fossil record of this lineage is represented by a single species (*Apis nearctica*) from the Miocene (13 MA) of Nevada^71^. The most likely route that the *Apis* lineage took to arrive in North America would have been via the Bering Isthmus^70^, which was present throughout the Neogene until ~5–7 Ma;^72^. This land connection would have allowed for the existence of expansive high-latitude terrestrial continuity, spanning the northern reaches of the Eurasian and North American continents. Thus *Apis* in North America may have originally inhabited this Arctic biome before dispersing southward into the mid-latitudes of North America. In which case, the polar *P*. *abstrusus* may have had opportunity to supplement its diet with honey.

**Figure 10:**
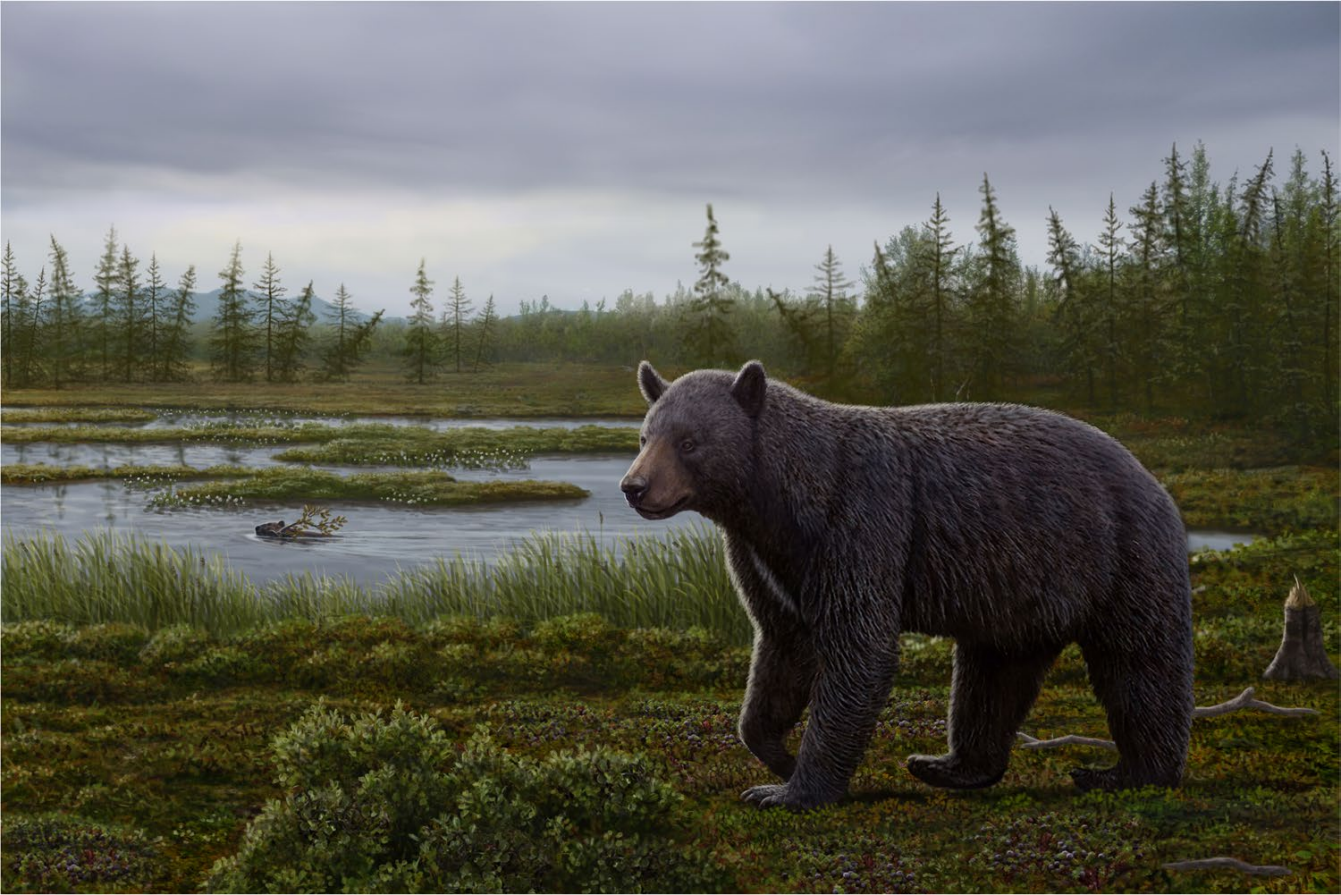
Reconstruction of the mid-Pliocene *Protarctos abstrusus* in the Beaver Pond site area during the late-summer. An extinct beaver, *Dipoides*, is shown carrying a tree branch in water. Plants include black crowberry (*Empetrum nigrum*) with ripened berries along the path of the bear, dwarf birch (*Betula nana*) in foreground; sweet gale (*Myrica gale*) carried by the beaver, sedges in water margins, flowering buckbeans along the mounds behind the beaver, and larch trees in distant background. Art by Mauricio Antón based on research of this paper and with input on plant community from Alice Telka.

Aside from the Beaver Pond site fossil bear, all other basal ursines are known from the northern mid-latitudes (30–40° N) of Eurasia and North America (Fig. 1, and Supplementary Information). The lack of fossil bears in the intervening latitudes reflects the scarcity of northern Neogene vertebrate fossil sites in these regions. Thus, the discovery of the Beaver Pond site *P*. *abstrusus* at 78°N fills a substantial geographical gap. The finding also shows that early ursines were adapted to northern forests with snowy winters. Moreover, the Beaver Pond site bear is a small-bodied bear with dental caries and associated with a polar forest, rich in seasonal fruits (Fig. 10), suggesting that the northern populations of *P*. *abstrusus* likely consumed large amounts of sugar-rich foods in the fall, a pattern consistent with preparation for hibernation seen in modern bears. If so, the Beaver Pond site bear represents the earliest known, and most primitive bear, to have hibernated. Modern ursid hibernators include high latitude/altitude Asian black bears (*U*. *thibetanus*), northern American black bears (*U*. *americanus*), all brown bears (*U*. *arctos*), and female polar bears (*U*. *maritimus*)^73^. Also, the fossil species of cave bears (*U*. *spelaeus* and *U*. *deningeri*) are inferred to have hibernated^74^. All living bears also employ a reproductive strategy of embryonic diapause (delayed implantation) with implicit adaptive value of reducing the cost of reproduction by truncating embryonic development and of optimizing birth season at the most appropriate time^75^. Furthermore, these reproductive cycles may regulate metabolism by facilitating earlier entry of pregnant females into winter-dormancy state^75,76^. In the context of the phylogeny of modern bears the northern *americanus-arctos-spelaeus-maritimus* clade appears to have acquired hibernation from a single ancestor. The case for Asian black bear is ambiguous because its nearest relatives are not known to hibernate; namely the sloth bear (*Melursus*) of India, the sun bear (*Helarctos*) of Southeast Asia. The early diverging spectacled bear (*Tremarctos*) of South America is also a non-hibernator (Fig. 6). If the northern adapted Beaver Pond bear was a hibernator, then hibernation can be traced to the ancestor of all modern bears. This would imply that the Asian black bear retains the primitive condition, and the Eurasian ancestor of the spectacled bear, which would have passed through cold Beringian habitat when it first immigrated to North America^77^, also employed hibernation as part of its repertoire for winter survival. In this evolutionary scenario modern non-hibernating bears, are interpreted to have secondarily lost this trait, in association with adaptation to warmer habitats.

## Methods

### Phylogenetic methods

Our phylogenetic analysis combined character matrices from Abella *et al*.^78^ and Qiu *et al*.^5^ and added several relevant basal ursines not present in either of the above authors (Table S1). All seven living ursine bears were included in the analysis. Our use of the term “ursine(s)” refers to the tribe Ursini, which include all taxa that fall within the clade of living sloth, sun, black, brown, and polar bears plus their fossil relatives to the exclusion of the tremarctine bears (Tremarctini). Together ursines and tremarctines constitute the subfamily Ursinae. Living ursids examined in this study include: *Tremarctos ornatus*, AMNH CA 2861, LACM 72530; *Melursus ursinus*, LACM 88916; *Helarctos malayanus*, LACM 52380; *Ursus thibetanus*, AMNH CA 1981, LACM 30781; *Ursus americanus*, AMNH CA 2886, AMNH CA 35005, LACM 92299; *Ursus arctos*, LACM 31257; *Ursus maritimus*, LACM 86096. Character coding and manipulation are done on Mesquite program^79^ and phylogenetic analysis is performed on TnT (version 1.1, Dec. 2013)^80^. Initial search resulted in a single shortest tree of 139 steps (tree search parameters: Implicit Enumeration and New Technology search; both methods yielded the same result). This tree has a number of nodes for living ursids that contradict molecular phylogeny. We then constrained our search using the topology of extant taxa, fixing the relationship based on nuclear DNA in Kutschera *et al*.^37^ and Kumar *et al*.^38^.

### Chronology

For estimates of magnetic ages we adopt the ATNTS2012 Geomagnetic Polarity Time Scale (GPTS) of Hilgen *et al*.^81^. Our usage of the Plio-Pleistocene boundary (Neogene-Quaternary boundary) follows the recent decision by the International Commission on Stratigraphy at the boundary of magnetochrons C2r-C2An (2.581 Ma)^82^.

### Surface scanning of skull

Skull elements of *Protarctos abstrusus* were scanned using an Arius 3-D laser scanner, digitally reassembled in PointStream software (Version 3.2.0.0)^83^ and converted into a triangulated polymesh surface using Paraform^84^. This model was later adjusted in Avizo 9.0^85^ by two of us (XW and SCW). Cranial measurements on the digital model were taken by tools provided in the above software.

### MicroCT scanning of teeth

MicroCT examinations were made of m1 and m2 from CMN 52078 A (left dentary), and left M2 from CMN 54380 using a SkyScan 1173 scanner operating at 70 kV, 114 microA and the images were reconstructed with an isotropic voxel size of 12.08941 micrometer for m1 and 10.30843 micrometer for m2. The reconstructed BMP images were imported into Fiji (V. 2.0.0-rc-30/1.49t) where they were reoriented to make the occlusal plane horizontal to the image edge, cropped to the size of the crown, contrast adjusted and converted to 8 bit tif files. The resulting images were imported into Avizo 9.0.0 Lite^85^ for analysis. The enamel and dentin were segmented and 3D models constructed. Cross-sectional images (oriented buccolingually) were made through regions of interest.

### Prevalence of caries in extant *U*. *americanus*

Data on prevalence of carious lesions in modern populations of American black bear (*U*. *americanus*) were collected from published data (see Table S3). Also the upper teeth of 57 specimens from northern populations from the collections of Canadian Museum of Nature were examined for the presence of caries: CMNMA 15004, 17790, 17791, 17933, 17953, 17958, 17959, 17970, 18038, 1826, 1830, 1831, 1833, 1834, 1836, 1840, 1841, 1842, 1844, 1905, 19598, 19816, 19817, 19818, 21811, 21812, 21813, 21814, 21817, 21880, 22009, 24245, 24247, 26695, 26696, 30874, 30875, 30876, 30877, 31764, 31765, 34109, 34335, 34336, 34337, 34338, 34339, 34340, 34341, 34342, 36926, 37352, 39744, A20682, A20683, A20684, 9577. Age class for the latter was determined from the fusion of cranial element^13^.

## Electronic supplementary material

Supplementary Information
