# Supplementary material for: A basal ursine bear (Protarctos abstrusus) from the Pliocene High Arctic reveals Eurasian affinities and a diet rich in fermentable sugars
Source: Sci Rep. 2017 Dec 18;7:17722. doi: 10.1038/s41598-017-17657-8 (PMC5735171; doi:10.1038/s41598-017-17657-8)
Supplement: Supplementary file 1 — Supplementary Information [file 41598_2017_17657_MOESM1_ESM.docx]

Supplementary Information for

**A basal ursine bear (*Protarctos abstrusus*) from the Pliocene High Arctic reveals Eurasian affinities and a diet rich in fermentable sugars**

Xiaoming Wang ^a,b,c,*^, Natalia Rybczynski ^d,e^, C. Richard Harington ^d^, Stuart C. White ^f^, Richard H. Tedford ^c,§^

^a^ Department of Vertebrate Paleontology, Natural History Museum of Los Angeles County, 900 Exposition Blvd., Los Angeles, CA 90007, United States

^b^ Key Laboratory of Vertebrate Evolution and Human Origins of Chinese Academy of Sciences, Institute of Vertebrate Paleontology and Paleoanthropology, Chinese Academy of Sciences, Beijing 100044, China

^c^ Division of Paleontology, American Museum of Natural History, Central Park West at 79th Street, New York, New York 10024, United States

^d^ Paleobiology, Canadian Museum of Nature, PO Box 3443 STN "D", Ottawa, Ontario K1P 6P4 Canada

^e^ Department of Biology & Department of Earth Sciences, Carleton University, 1125 Colonel By Dr, Ottawa, ON K1S 5B6, Canada

^f^  School of Dentistry, University of California, Los Angeles, 10833 Le Conte Ave., Los Angeles, California 90095, United States

^§^ Deceased

* Corresponding author

This PDF file includes:

1. Institutional Abbreviations
2. Description of fossil skull material
3. Comparison of North American Records of fossil ursines
4. Remarks on Eurasian early ursines
5. Ursine phylogenetics, a background

Table S1. Cranial measurements of basal ursine bears

Table S2. Dental measurements

Table S3. Dental cary prevalence in *Ursus americanus* populations

Table S4. Dental cary prevalence in northern *Ursus americanus* by age class

Table S5. Descriptions of characters used in the data matrix

Table S6. Data matrix for phylogenetic analysis

Figure S1. Beaver Pond site excavations

Figure S2. Upper molar proportions in ursines

Figure S3. Anterior (A) and posterior (B) views of skull of *Protarctos abstrusus*

Figure S4. Stereo photos of left basicranial region of *Protarctos abstrusus*, CMN 54380, ventral view.

Figure S5. Stereo photos of left petrosal of *Protarctos abstrusus*, CMN 54380, ventral view

Figure S6. Right dentaries of *Protarctos abstrusus*

SI References

1. **Institutional Abbreviations**:

AMNH, American Museum of Natural History, New York, New York, USA

CMN, Canadian Museum of Nature, Ottawa, Ontario, Canada

HMV, Hezheng Paleozoological Museum, Hezheng County, Gansu Province, China

IVPP, Institute of Vertebrate Paleontology and Paleoanthropology, Chinese Academy of Sciences, Beijing, China

LACM, Natural History Museum of Los Angeles County, Los Angeles, California, USA

NUFV, Nunavut Fossil Vertebrates, currently housed at CMN

UMMP, University of Michigan Museum of Paleontology, Ann Arbor, Michigan, USA

USNM, United States National Museum of Natural History, Smithsonian Institution, Washington, D.C., USA

1. **Description of fossil skull material**

**Skull** (Figs. 3-6; supplementary Figs S3-S5, Table S1). Individual pieces of the skull are often quite small and their different colorations suggest different degrees of exposures to UV light. Probably due to a lack of sediment compaction, distortion to individual pieces of bones are minimal and many of the fragments greater than 1 cm in diameter could be glued, but numerous smaller pieces could not easily placed in their proper positions. This has yielded five major skull components. Sufficient contacts between three of these pieces are present permitting a reasonably accurate digital reconstruction of much of the skull based on laser scanned 3D images (Figs. 3-6).

In anterior view (Supplementary Fig. S3), the anterior naris is relatively large and wide. The forehead is not domed between the postorbital processes, but is flat and does not obscure the post temporal crests, as does in most living ursines. The above features are closest to those in the Indian sloth bears. In lateral view (Fig. 2), the skull is not very dolichocephalic and the rostrum is far from being lengthened as in advanced living ursids such as the American black bear-polar bear clade. The most noticeable feature is the relatively flat forehead in profile view in contrast to all living ursines, plus *Ursavus tedfordi*, that have different degrees of inflation of the forehead due to enlargement of the frontal sinus. As a result, the top surface of the skull shows no upward doming as is seen in nearly all living bears. Both nasals are nearly complete with anterior margins intact. The anterior part of the nasals is slightly widened, but overall the lateral edges of the nasals are largely parasagittal in orientation. The posterior segment of the lateral margins of the nasals narrows toward the median plane and extend caudally beyond the anterior rim of the orbit. The lateral sutures for the nasal process of the premaxillary is obscured by fusion, but faint outlines seem to indicate a strong posterior process, possibly as wide as 8-9 mm, extending posteriorly to meet the frontal process just behind the anterior edge of the infraorbital canal. The nasal process of the frontals penetrates between maxillary and nasal ending anterior to the anterior orbital rim. The dorsal surface of the frontal between the postorbital processes is rather flat in anterior view. The postorbital process bends downward, and its inner surface is hollow and partitioned by a bony septum. The temporal crest leading from the postorbital process is not prominent, just slightly raised above the skull roof. Toward the midline, the left temporal crest shows lesions, possibly due to healed injuries incurred during life (Fig. 3). The temporal crests converge about 5 cm posterior to the postorbital process and unite to form the sagittal crest. The location of the postorbital constriction is not preserved. A prominent sagittal crest, as high as ~20 mm about 30 mm in front of the inion, extends caudally such that the inion overhangs the occipital condyles by more than 10 mm. At the highest point of the sagittal crest is a broad, scalloped 25 mm notch, again probably due to injuries during life, followed behind on the left side by a long, nearly vertically-oriented lesion. Rough surface textures indicate healing after injury. As in the sagittal crest, the lambdoidal crest also extends posteriorly to converge on the inion.

The anterior margin of the infraorbital canal (Fig. 2, Supplementary Fig S3) is about 2 mm behind the anterior edge of the M1. The jugal has a prominent postorbital process, with a maximum depth of about 28 mm. The zygomatic arch has a minimum depth of 24 mm near the lowest point of the orbital rim. Posterior to the postorbital process, the zygomatic process of the jugal sharply narrows to a minimum depth of 10.5 mm, and about 4 mm behind this point, the suture of the squamosal process of the zygoma is visible. Lacking the anterior process of the squamosal, it is not possible to know if a steep elevation of the squamosal occurs immediately behind the narrowing of the zygomatic arch, as is seen in *Ursavus tedfordi* and several living ursines.

In posterior view (Supplementary Fig S3), the lambdoidal crests have a concave profile between the top and mid portion, at which point there seems to be a constriction, although damage by rodent gnawing to this area on both sides prevents a definitive assessment. The supraoccipital shield has a prominent keel, as high as 5 mm, along the mid line. There are additional rodent gnaw marks on both occipital condyles, more so on the left side.

The palate is largely missing (Fig. 3) except the anterior portion in front of the P4 on the right side. The posterior extent of the incisive foramen is also missing. It is not possible to estimate how far the palatine extends behind the M2.

The basicranial area on both sides is partially preserved but the medial portion of both bullae and the caudal entotympanic is missing (Fig. 3; Supplementary Fig. S3-4). The bulla is uninflated, typical of type A bulla in basal arctoids ^1^. There is, however, a prominent medial ridge along the parasagittal plane on the ventral surface of the bullae, best seen on the right side that begins at the anterior carotid foramen and ends posteriorly toward the paroccipital process, although the exact end is missing. To the medial side of this ridge is the suture zone for the attachment of the rostral entotympanic and a triangular suture surface is visible on both sides, but the rostral entotympanic bones are missing. The external auditory meatus extends laterally about 12-13 mm lateral of the postglenoid foramen. The mastoid processes are incomplete on both sides but that on the right side is better preserved. The ventral and lateral faces of the right mastoid process show signs of incomplete ossification and a cartilaginous cap is presumed to exist there during life. The mastoid process is moderately enlarged, but not as much as in advanced living ursids such as the American black bear – polar bear clade, and does not expand ventrally beyond the lowermost point of the paroccipital process. The paroccipital process is ventrally oriented and flattened to form a spatulate anterior facet. Its contact with the posteromedial aspect of the bulla is missing but can be inferred to be a relatively narrow strip on the medial aspect of the process. An alisphenoid canal is present on both sides, but bones around its anterior opening are not well preserved to show its relationship with the foramen rotundum. The posterior opening of the alisphenoid canal is within an elongated pit shared with the foramen ovale. Immediately behind this pit is the opening of the Eustachian tube.

Both petrosals are also preserved in isolation (Supplementary Fig. S5). On the ventral surface of the promontorium, there are 2-3 transpromontorial grooves forming parallel arches coursing through the apex of the promontorium (Fig. 8). These grooves are presumably left by impressions of a small side branch of the caroticotympanic arteries and nerves as also seen in modern raccoons ^2:fig. 83^.

**Lower Jaw** (Supplementary Fig. S6). The coronoid process is missing on both jaws, although the basal part is still intact on the right side. The anterior border of the ascending ramus almost touches the posterior edge of the m3 root. The masseteric fossa is deep, leaving strong impressions of muscle attachment scars by temporalis and masseter. The lower rim of the masseteric fossa forms a distinct ridge lower masseteric crest of ^3^ that converges posteriorly toward the lower surface of the horizontal ramus. There is no pre-masseteric fossa, although a very shallow, vague, and ill-defined depression can be felt when running fingers across it. Both angular processes are missing. A distinct scar for digastric muscle marginal process of ^3^ runs along the ventromedial surface of the horizontal ramus and terminates either at (right), or slightly behind (left), the mandibular foramen.

The horizontal rami (depth on the medial side at m1 talonid is 36.4 mm and width at same position is 14.0 mm) are modestly strong. The lower border is straight and begins to curve upward posterior of the m3. There is a slight chin at the ventral end of the symphysial suture. Four mental foramina are seen on both jaws, the anterior one being either in front of or at the p1 and the posterior one being at the junction of p4-m1.

**Upper Teeth** (Figs. 2-5; Supplementary Table S2). Only the left and right I1s are preserved. Heavy wear on their crowns has obscured cusp morphology. The I2s and I3s are missing and have left progressively larger roots. The alveoli of the I2s are mediolaterally compressed whereas those of I3s are more rounded forming an oval cross section. There is a 5 mm diastema between the alveoli of I3 and C. Only the right upper canine is preserved. It sustained minimum wear at the tip. The root of the C is very strong, reaching backward to the level of anterior P4. The cross section at the enamel-dentine junction is oval-shaped. There is a thin but distinct ridge on the posterolateral aspect of the upper canine running along the entire length of the crown.

P1-3 are missing. Consistent with a rostrum that is not elongated, the premolars, judging by their alveoli, are not extremely reduced as in the American black bear-polar bear clade and they are not spaced out by long diastema. The single P1 root is coalesced with that of the canine, and the anterior end of the P1 was presumably in contact with the canine. The mediolateral diameter of the P1 alveolus is 4.4 mm in contrast to 2.9 mm for that of P2, indicating an enlarged P1 relative to P2 seen in many ursines. P2 has a single root, which has a rounded cross section. P3 has two roots. The anterior alveolus has a maximum anteroposterior dimension of 5.4 mm and 2.9 mm in transverse diameter, and its posterior alveolus is somewhat smaller with a transverse diameter of 2.6 mm.

The carnassial, P4, has three roots. A narrow but distinct cingulum surrounds the entire tooth except the protocone. The paracone is the largest and tallest cusp, occupying about 2/3 of the entire tooth. There is no parastyle. An indistinct anterior ridge runs along the entire paracone. A distinct carnassial notch separates the paracone and metastyle. The metastyle is about half as tall as the paracone. Although both paracone and metastyle are mostly cone-shaped, there is a short carnassial blade running along the medial side of the cusps and its wear facets are straight and aligned along a single plane. A small protocone is located on the posterior third of the tooth, and its apex is slightly anterior to the carnassial notch, although its anterior and posterior bases bracket the carnassial notch. It is also low-crowned with its apex slightly above the anterior cingulum.

The first molar is enlarged relative to the carnassial. With a quadrate outline, the M1 has a thin cingulum surrounding much of the tooth. The lingual cingulum is relatively strong between protocone and metaconule. The protocone and metacone are nearly identical in size, with the former slightly taller-crowned. There is a small but distinct metastyle but no parastyle is present. The protocone-postprotocrista-metaconule forms a nearly straight crest about half as tall as the paracone. A large wear scar is present between protocone and paracone and is the only enamel surface that has been worn down to the underlying dentine. Enamel wrinkles radiate from the metacone and metaconule, but those along the surface of protocone and paracone are polished off by wear.

There are four roots on M2, one above the paracone, metacone, and coalesced protocone and metaconule, and a fourth tiny root lies between paracone and protocone seen only on the right side. The M2 is elongated relative to the M1 due to a posteriorly-expanded talon. M2 cingulum is less distinct than that of the M1. Its paracone and metacone are of equal size but are lower-crowned than their counterparts in M1. There is a hint of a parastyle, but wear in this area does not permit positive recognition. As in M1, the preprotocrista-protocone-postprotocrista-metaconule forms a long ridge along the entire length of the tooth. In lateral view, the talon bends dorsally, presumably to occlude with a forwardly tilted m3. In occlusal view, the talon narrows toward the posterior end, especially along the labial edge of the tooth. Distinct wrinkles are present throughout the occlusal surface. A large, rounded pit is at the posterior end of the talon dropping steeply into the underlying dentine leading toward the pulp cavity. The enamel edges surrounding this pit are serrated but the dentine surface is smooth.

**Lower Teeth** (Fig. 6; Supplementary Figs. 6, Table S2). Lower incisors on both sides are lost, as are their alveoli. Both canines are well preserved. Their roots are robust and thicken in midsection, forming a strong anchor for the crown. The enamel-dentine junction is oval in outline. There is a very faint ridge in the anteromedial aspect of the tooth and one in the posterolateral aspect as well. The p1-3 are all missing. The total length of p1-m3 is ~82 mm and that for m1-3 is ~50 mm. The p1 alveoli on both sides indicate a root of equal diameter as that of the p2 (left) or slightly smaller (right). The p2 is also single-rooted and its alveoli are oval in outline with its long axis in anteroposterior direction. The p3 has two roots on both sides. Those on the right side are much smaller than on the left side, and show signs of having been lost shortly before death and the root canals were well on their way to being healed.

The p4 has a narrow cingulum surrounding the entire tooth, and at anterior and posterior ends, there is vague bulging to indicate a faint cingular cusp. The p4 main cusp is low-crowned and there is a very weak anterior and posterior ridge leading down from the apex. A tiny posterior accessory cusp is present immediately on the lingual side of the posterior ridge. This cuspule, and its variations, also occurs in the Polish *U. minimus* ^4^ and some advanced ursines such as *Ursus arctos* and *U. deningeri* ^5:fig. 1,6^.

The lower carnassial, m1, has sustained extensive wear such that some cusp morphology is less reliably identified. A weak labial cingulum is present along the anterior and posterior portion of the trigonid as well as the talonid. The trigonid is about twice as long as the talonid. The paraconid bends toward the lingual side, although wear on this cusp does not permit observation of the carnassial blade. The protoconid is the largest cusp of the tooth and its base swells toward the lingual side. The metaconid is located at the posterolingual aspect of the protoconid. There is no pre-metaconid, nor is there a low crest extending forward as in more advanced ursines. The posterior face of the trigonid suffered from extensive wear, particularly on the metaconid, and presence or absence of very faint ridges, important characters according to Qiu et al. ^7^, are not clearly observable. However, a small cuspule at the base of the protoconid, at the posterior aspect, is seen just in front of the valley dividing trigonid and talonid. The talonid is dominated by a large hypoconid, occupying two-thirds of the talonid, although this cusp suffers from extensive wear on both sides. On the lingual side, there are two small cusps, the pre-entoconid and entoconid. The talonid basin may have been partially enclosed by a posterior ridge of the hypoconid, but damage to this area makes this observation less secure. The talonid is slightly wider than the trigonid, but it lacks a prominent labial expansion seen in some ursids.

The m2 is modestly enlarged but still shorter than m1, and the trigonid is of about the same length as the talonid. Extensive wear on the occlusal surface prevents clear observation of cusp morphology. The protoconid is larger than metaconid, but their relative height is not observable due to wear. Both cusps have a triangular base as shown by the worn-down enamel edges. Two small cuspules are visible immediately in front of the metaconid along the lingual rim and whether or not additional cuspules are present along the anterior rim is not possible to ascertain. Behind the protoconid is a crest that connects with the hypoconid, and there is a hint on the right m2 that a small cuspule may have been present at the base of this crest, although such a cuspule is not seen on the left m2. The posterior surface of the trigonid appears to be smooth but this can be due to wear. There is an indistinct notch between the protoconid crest and hypoconid. The hypoconid is the largest cusp on the talonid, occupying about two-thirds of the space. Heavy wear on this cusp does not allow detailed observation. A longitudinal crest trails behind the metaconid, and two discrete cuspules are clearly identifiable along this crest. Caudal to these cuspules is the entoconid crest, which has a faint swelling at the base suggesting a cusp on the left m2 whereas two small cuspules are possibly present on the right m2. The entoconid crest continues caudally to form a posterior rim that connects to the hypoconid such that the talonid basin is closed caudally.

The m3s are missing on both jaws with the left alveolus being more complete. The cross section of the single root from left m3 narrows toward its posterior end. Judging by the alveolus length, the m3 is approximately 13-14 mm in length, in line with relative length to m2 in *Protarctos yinanensis*.

1. **Comparison of North American Records of fossil ursines**

Bjork ^8^ named an extinct bear, *Ursus abstrusus*, based on a right dentary fragment with an m1 plus alveoli of p4 and m2-3 (UMMP V53419, locality UM-Ida 79-65), plus an edentulous premaxillary-maxillary fragment (USNM 24932), from the Glenn’s Ferry Formation, southwestern Idaho (red star 2 in Fig. 1). An additional distal left humerus (UMMP V49950) was initially referred to “Tremarctinae gen. et sp. indet.” because of its possession of an entepicondylar foramen ^8: 18^. Ruez ^9,10^, however, argued that this character is also present in early ursines from Eurasia and thus conservatively referred UMMP V49950 to *U. abstrusus*. Locality UM-Ida 79-65 was at ~3,220 ft level of Section H near Hagerman, 60 ft above a volcanic ash marker "Tga1" in ^8^. The fossil assemblage was broadly referred to as the Hagerman Local Fauna by Bjork, which is radiometrically constrained within two volcanic layers dated to 3.48 Ma (Deer Gulch Lava) and 3.75 Ma (Peter Gulch Ash), as well as by a reversed magnetic polarity ^11^, in middle Blancan North American Land Mammal age Blancan III of ^12^. The Beaver Pond site, dated using terrestrial cosmogenic nuclide dating ^13^ is thus about the same age as the Hagerman Local Fauna.

Gustafson ^14^ listed *Ursus* cf. *abstrusus*, consisting of fragments of a right M1 and left m1, from an uncertain level of the Ringold Formation in south-central Washington and placed these within the White Bluffs Local Fauna. Tedford and Martin ^15^ subsequently referred these teeth, plus additional materials from the Ringold Formation, to their newly named tremarctine bear, *Plionarctos harroldorum*. Another possible Pliocene occurrence of *Ursus* in North America was recorded by Johnston and Savage ^16^ from Cita Canyon, Texas, which straddles the Plio-Pleistocene transition at the Gauss/Matuyama boundary (2.6 Ma), but no description was provided. However, Kurtén ^17^ questioned whether or not this is an ursine. With the re-assignment of Ringold specimens to the tremarctines and other Blancan materials questioned, *Ursus abstrusus* from the type locality remains the only true ursine bear in the Pliocene of North America see also ^18^ before the discovery of the Beaver Pond bear.

Dental dimensions of the holotype of *Protarctos abstrusus* are very similar to those of the Beaver Pond bear, with m1 length being almost identical and m1 talonid width somewhat wider (Supplementary Table S2). Its m2 alveoli also suggest a similar size relationship to m1 as that in the Beaver Pond jaws. Given that the age of the Hagerman bear is nearly identical to that of the Beaver Pond site (see above), initial reference of the Beaver Pond bear to the Idaho form by Harington ^19,20^ and Tedford and Harington ^21^ is judicious until additional materials are discovered from the Glenn’s Ferry Formation. Tedford and Harington ^21:388^, however, remarked that the Beaver Pond bear appears “more primitive than those known in Eurasia.”

Despite his befuddlement by the meager materials from Idaho, as is apparent in his chosen species name of *abstrusus*, Bjork ^8^ pointed out that the Hagerman bear is at nearly the same stage of evolution as *Ursus boeckhi* ^22^ (see below for additional discussion), an insight born by our own observations as well. However, possibly influenced by his contemporaneous European colleagues, Bjork ^8:17^ speculated that “*Ursus americanus* may have speciated in North America after the migration of *U. abstrusus*,” despite his acknowledgment of the “differences in timing and placement of phylogenetic connections.” In his preliminary list of vertebrate taxa from the Beaver Pond site Harington ^19,20^ mentioned “*Ursus abstrusus –* primitive black bear.”

1. **Remarks on Eurasian early ursines**.

In Europe, a rich literature exists on fossil bears going back almost 200 years. Despite such a long history of study, however, considerable controversies still exist regarding the taxonomy of Eurasian Pliocene ursine bears see summary of early literatures in ^6^. Central to the initial evolution of ursines is *Ursus boeckhi* ^22^ originally established based on isolated cheek teeth from Baróth-Köpecz lignite beds of Romania (previously Hungary) (red triangle in Fig. 1) in the early Pliocene (European MN14 or 15). Schlosser recognized the basal status of *U. boeckhi* and compared it with Miocene *Ursavus brevirhinus* and *Ursavus primaevus*. His illustration shows that the lower cheek teeth of *Ursus boeckhi* has a simple m1 morphology that has no pre-metaconid, pre-entoconid, and zigzag groove on trigonid, as well as a simple m2 with few accessory cuspules ^22:pl. 12, figs. 3-8^. Maier von Mayerfelds ^23^ figured additional skull fragments with entire left dentition from the type locality. Subsequently Kretzoi ^24^ erected a new genus, *Protarctos*, for the Romanian form in recognition of its primitive status. Later students tend to agree on its basal position and maintained *Protarctos boeckhi* as a distinct species ^4,6-8,25^. Morlo and Kundrát ^26^, however, adopted a highly inclusive *U. minimus* that subsume the Romanian form as a subspecies, *U. minimus boeckhi*, in which they also questionably included the North American *U. abstrusus*. More recently, Wagner et al. ^27^ lumped all European ursine bears from the Early Pliocene into *Ursus* gr. *minimus*-*thibetanus*, following a similar scheme by Mazza and Rustioni ^28^.

Possibly at a similar stage of dental evolution is *Ursus ruscinensis* (initially named as a subspecies of *Helarctos arvernensis*) from the Fort Serrat d'en Vacqner near the southern French town of Perpignan ^29^. However, as noted by Qiu et al. ^7^, this species does seem to have some peculiarities of its own including its deep but blunt angular process ^29:pl. III^. To the above two European species, Qiu et al. ^7^ also added the Chinese *Ursus yinanensis* Li, 1993 and together they were placed under the genus *Protarctos*, a decision followed in this study.

*Protarctos abstrusus* falls within the general dental plan of *P. boeckhi* and *P. ruscinensis*, such as a simple m1 trigonid without a pre-metaconid; nor is there a lingual cingulum along the trigonid that is present in slightly more derived forms in Europe (see below). However, the North American *P. abstrusus* and East Asian *P. yinanensis* share the presence of two lingual cusps in m1 talonid, pre-entoconid and entoconid, in contrast to a single entoconid cusp in *P. boeckhi* and *P. ruscinensis*. Tedford and Martin ^15:fig. 4^ illustrated this character in *U. abstrusus*, contrasting it with the primitive tremarctine *Plionarctos harroldorum*. This double entoconid in the Asian and North American Pliocene *Protarctos* appears to be a retention of a primitive condition as it is present in various stem species in *Ursavus* and *Ballusia* ^3^, although sun bears and polar bears also variably possess this condition ^6:plates V-VIII^. If this is the case, the Asian and North American *Protarctos* is a small step more primitive than their European counterparts (see Fig. 7).

Another basal ursine widely cited in the discussion of evolution of black bears is *Ursus minimus*, initially named on a partial crushed skull with left and right upper teeth from Ravin des Etouaires near Perrier in southcentral France ^30^. In the following year, Croizet and Jobert ^31^ named another species *Ursus arvernensis* based on a partial skull and upper teeth from Puy-de-Dôme, which was close to the type locality of *Ursus minimus* and most researchers seem to agree that they are the same species ^6,7,26,28,32^. In addition to Perrier, other European sites are also known to produce *U. minimus*, such as Węże and Rębielice Królewskie-Kamieniołom (Poland), Wölfersheim (Germany), Odessa Catacombs (Ukraine), Layna (Spain), and Osztramos (Hungary) ^7,27,33^. Erdbrink ^6^ suggested that *U. minimus* is almost indistinguishable from Asian black bear *U. thibetanus* in dental morphology, an idea that was followed by some recent workers ^28^. Others would refer directly to *Ursus thibetanus*, such as an early Pliocene (MN 15) tooth from Moldova ^34^ or adopt a nomen of *Ursus* ex gr. *minimus* or *Ursus* ex gr. *minimus-thibetanus* ^27,35-37^. In northern Asia, *Ursus minimus* has been listed in the Pliocene Udunga locality (Udunginian Faunal Complex) in the Temnik River valley of the Transbaikal region, Siberia ^38,39^, although no formal description of a fragmentary upper and lower jaw is currently available (Sotnikova, pers. comm.).

Li ^25^ described a new species, *Ursus yinanensis*, based on a partial skull and jaws (IVPP V10320) from a cave in Yinan County, Shandong Province (red circle 3 in Fig. 1). The Shuangquanxi Mountain Cave (IVPP locality 88002) deposit is largely reddish brown clays and limestone breccia, with a minimum thickness of 4.2 m. Li ^25^ suggested a “middle Pliocene” age for the cave sediments, largely based on the stage of evolution of *U. yinanensis*, postulated to be between the European *Ursus boeckhi* and *U. minimus*, an age assessment that is consistent with the presence of a new cricetid rodent, *Kowalskia yinanensis*, from the same cave ^40^. Subsequent European authors, however, all dismissed *Ursus yinanensis* as a synonym of the European *Ursus minimus* ^26,33,41,42^, despite Li’s ^25^ explicit statement that *U. minimus* is larger in size, with a pre-metaconid on m1, and having larger and more complex molars when compared to the former. More recently, Qiu et al. ^7^ described additional materials of *U. yinanensis* from the Longdan Fauna in the early Pleistocene loess deposits in Linxia Basin, Gansu Province (red circle 4 in Fig. 1) ^43^. Once again Qiu et al. ^7^ reasserted the more primitive status of the Chinese species, such as its lack of an m1 pre-metaconid and smooth posterior face of the m1 trigonid in the Linxia Basin materials, thus reconfirming the validity of *U. yinanensis* as well as extending its range into the early Pleistocene. Moreover, Qiu et al. did not consider the Chinese and European forms to belong in the same genus. Instead, they placed the Chinese *yinanensis* in the genus *Protarctos* and included the European *minimus* in *Euarctos*, largely based on Heller’s ^44^ observation that *E. minimus* from Perrier possessed a small pre-metaconid and V-shaped ridge on posterior face of m1 trigonid, derived characters used by Qiu et al. to define the advanced status of *Euarctos*. As shown in Fig. 7, we confirm Li’s (1993) and Qiu et al.’s (2009) observations that *P. yinanensis* is somewhat less advanced than *E. minimus* in dental proportions. It is, however, apparent that Qiu et al.’s concept of these genera are all based on a notion of stage of evolution (largely dental) and as such are paraphyletic except for the terminal taxon *Ursus*.

Another Chinese Pliocene ursine is a left jaw from Yushe Basin, Shanxi Province referred to *Ursus* sp. by Teilhard de Chardin ^45^, which Erdbrink ^6^ thought to be part of his *Ursus thibetanus* complex, although its m2/m1 ratio (85%) falls in the *Protarctos* or *Euarctos minimus* range. Teilhard de Chardin did not describe this specimen, but his figure ^45:fig. 14^ seems to suggest the presence of a pre-metaconid on m1, indicating a more advanced status than *Protarctos*. Zdansky’s ^46^ *Ursus* cfr. *boeckhi* from Liang-Chia-Ho in Shanxi Province may also be a Pliocene record (Qiu and Tedford, unpublished ms.), which was later recognized as belonging to a fossil species of Malayan sun bear *Helarctos sino-malayanus* ^47^.

By the Pleistocene, most Chinese records were of true black bear grade, such as that of early Pleistocene Nihewan ^48^, early Pleistocene Huiyü (“Zhoukoudian” Locality 18 in Mentougou area, southwest of Beijing) ^45^, Zhoukoudian Locality 1 ^49,50^, Yan-ching-kao ^51^, Renzi Cave ^52^, etc. Most of these were either *Ursus thibetanus* or closely related forms ^6^, or isolated records of sun bears from south China ^53^.

1. **Ursine phylogenetics, a background**

In recent years there is considerable interest in the molecular phylogeny of living ursids. That black bears are in a more basal position relative to the cave-brown-polar bear crown clade is generally borne out by recent studies ^54-66^, as has long been suggested by morphologists ^4,28^.

The detailed relationships, particularly among black bears, are still controversial with no sign of convergence. In early studies of mitochondrial DNAs, a close relationship of American black bear, *Ursus americanus*, with the Southeast Asian sun bear, *Helarctos malayanus*, has been postulated ^67^. Increasingly, a sister-taxon relationship between the Asian and American black bears is evidenced by additional sequence of the mtDNA ^60,68,69^, with the sun bear as a sister to the American and Asian black bear clade, as also supported by some morphological assessment that points to Asian black bears as being ancestral to American black bears ^28,70^. The addition of select regions of nuclear genome and Y chromosome genes, however, revealed additional complexities ^63-65^. Most recently, analyses of mtDNA, nuclear genes, and Y chromosomes, plus increased sampling of additional individuals, have led to the recognition that incomplete lineage sorting and introgressive hybridization may be responsible for the gene tree discordance and after controlling for such effects, *U. americanus* is back in the brown-polar bear clade and Asian black, sun, and sloth bears form a clade of their own ^66,71^.

Despite the attention paid to fossil ursids, there is no comprehensive cladistic analysis on either fossil or extant ursines until quite recently. While analyzing relationship of the giant panda clade (Ailuropodinae), Abella et al. ^72^ first proposed a cladogram based on a 19 taxa by 82 character matrix that includes all living ursids. More recently, Qiu et al. ^3^ performed an analysis of a narrower scope, adopting only about a quarter (20 of 82) of Abella et al.’s characters and dropping all extant taxa, targeting, instead, the basal ursine part of the phylogeny.

Qiu et al. ^7^ proposed a taxonomy of Ursinae largely based on their notion of m1 developments. At the base of their scheme is the genus *Protarctos*, including *P. boeckhi*, *P. ruscinensis*, and *P. yinanensis*, with such plesiomorphic conditions as m1 lacking a pre-metaconid and pre-entoconid, smooth posterior face of m1 trigonid (it is unclear where Qiu et al. place the living sloth and sun bears, but it seems that they are inclined to place these in a more basal position due to the relatively short molars of the sloth and sun bears). At an intermediate stage of evolution is the genus *Euarctos*, which contains *E. pyrenaicus* and *E. minimus* plus two extant black bears, *E. thibetanus* and *E. americanus*. The m1s in this group possesses a pre-metaconid and V-, Y-, or X-shaped ridges on posterior face of trigonid, but still lacking a pre-entoconid. The most derived genus is *Ursus*, including *U. etruscus*, *U. spelaeus*, *U. arctos*, and *U. maritimus*, and has acquired “bunodont” m1 pre-metaconid and pre-entoconid. Whereas these characters certainly constitute part of the dental evolutions, they are just as likely to be homoplastic as seen in many other dental characters. For example, the pre-entoconid (or “double entoconid”) is known to occur early in ursid phylogeny ^3:char 32^, disappear at the base of ursines, and then reappear again in polar bear and in some sun bears ^6^. Even if these characters were completely consistent, the taxonomy derived from them is almost certainly about evolutionary grades, not monophyletic clades (Fig. 7).

**Table S1**. Cranial measurements (mm) of basal ursine bears; see definitions in Qiu et al. ^3,7^.

**Table S2.** Dental measurements (mm). Data for extant ursines (*Melursus ursinus*, *Helarctos malayanus*, *Ursus thibetanus*, *Ursus americanus*) are adopted from Erdbrink ^6:tables II, III, IV^; only specimens with a complete set of P4-M2 measurements are used.

**Table S3.** Dental caries prevalence in *Ursus americanus* populations.

| **Region** | **source** | **N** | **Number with caries** | **% with caries** | **Source** |
| --- | --- | --- | --- | --- | --- |
| Alaska | CMN coll. | 8 | 0 | 0 | This study |
| Alaska | MVZ coll. | 57-59* | 1** | 2** | ^73^ |
| Yukon | CMN coll. | 28 | 4 | 14 | This study |
| Northwest territories | CMN coll. | 9 | 4 | 44 | This study |
| Northern Quebec, Newfoundland & Labrador | CMN coll. | 12 | 4 | 33 | This study |
| British Columbia | MVZ coll. | 32-34* | 4** | 12-13** | ^73^ |
| Michigan | live | 35 | 7 | 20 | ^74^ |
| Wisconson | live | 86 | 9 | 11 | ^74^ |
| California | MVZ coll. | 71-73* | 0** | 0** | ^73^ |

*Hall 1940 (published April 1940) used a sample size of 195, but did not provide specimen numbers. The Museum of Vertebrate Zoology, University of California, Berkeley ARCTOS database (<http://mvz.berkeley.edu>, <http://arctos.database.museum>, accessed 2016.11.09) shows 197 *Ursus Americanus* specimens, collected 1939 or earlier. The ARCTOS derived list of 197 specimens was used to estimate the number of specimens per region that Hall likely included in the 1940 publication. The three regions with the largest sample sizes are shown in this table: Alaska, British Columbia and California.

**These might be underestimates. Hall 1940 provided photographs of specimens identified with carious lesions. Notably all figures show lesions that are very large, with gross pathologies at very advanced stages. Presumably these bear populations would have included individuals with smaller caries, representing earlier stages of carious development. Small caries would have been less visible, and could be more easily missed. From the figures provided, it seems Hall’s (1940) study was inadvertently biased to include only the most advanced cases, implying that the results are underestimates.

**Table S4**. Dental cary prevalence in northern *Ursus americanus* by age class. Sample from Canadian Museum of Nature and regions represented include Alaska, Yukon, Northwest Territories, northern Quebec, Newfoundland & Labrador.

| **Estimated age (yrs)*** | **N** | **Individuals with caries** | **% with caries** |
| --- | --- | --- | --- |
| <2 | 8 | 0 | 0 |
| 2 – 5 | 32 | 3 | 9 |
| >5 | 17 | 9 | 53 |
| **Total individuals:** | **57** | **12** | **21** |

*Age was estimated based on criteria determined by Marks and Erickson ^75^, as follows: 1) Specimens where the suproccipital-basioccipital and exoccipital-supraoccippital are open are less than two years old; 2) Fusion of the basioocciptal and basisphenoid suggests the individual died when it was over 5 years of age.

**Table S5.** Descriptions of characters used in the data matrix (Table S6).

There are two recent phylogenetic analyses on fossil ursids. Whereas both are limited analyses focusing on phylogenetic regions of interests, that by Abella et al. ^72^ has a more extensive character coverage, a total of 82 characters including cranial characters, as well as a greater sampling of taxa (10 extinct and 9 extant). That by Qiu et al. ^3^, on the other hand, focuses on the transition to basal ursines, which excludes living ursids (as well as several fossil pandas) from analysis but includes a few transitional species of *Ursavus*. The latter also eliminated cranial characters but added many dental characters. Given incompatibility of the two matrices, the matrix by Abella et al. is more appropriate for our purpose because it includes extant clades that encompass regions of interest to this study. We do, however, attempt to incorporate, to the extent appropriate, Qiu et al.’s additional taxa and characters except two of their taxa with extensive missing data, *Ursavus intermedius* and *Ursavus ehrenbergi*. The character descriptions below largely follow those by Abella et al. ^72^ with appropriate notes of how we or Qiu et al. ^3^ differ from one another. While we strive to independently code characters, many taxa more basal to the ursines are minimally relevant to the present analysis and their coding is not always verified. As a result, inconsistency between Abella et al. and Qiu et al. can be difficult to reconcile.

There are several instances in which Abella et al. (2012) had coded redundant characters (characters that are correlated with each other or described in different ways in others), such as their character 27 (blade-like vs. bunodont m1 paraconid) and character 30 (blade-like vs. bunodont m1 protoconid), characters 49 and 51, characters 23 and 56, 10 and 2, and characters 24 and 57. These are combined in the present study. We have also dropped characters, as did in Qiu et al. (2014), that are either autapomorphies (characters 18, 26, 72 in Abella et al., 2012), not variable (character 21 in Abella et al., 2012), self-contradictory (character 28 in Abella et al., 2012), correlated to others (characters 27 and 34 in Abella et al., 2012), uninformative in the ursine clade (characters 7, 8, 13, and 20 in Abella et al., 2012), ill-defined (characters 36, 40, 41, 42, 43, 44, 63, 74, and 82 in Abella et al., 2012), or failed to verify in specimens we have examined and/or difficult to define (characters 12, 14, 15, and 64 in Abella et al., 2012).

It is also less than optimal to use living *Canis lupus* as an outgroup by Abella et al. (2012), which is too far removed from the basal ursid to accurately reflect the ancestral conditions. Qiu et al.’s (2014) use of *Cephalogale minor* is thus a better choice. However, despite its being frequently cited as a basal ursid, based on many jaws from “Phosphorites du Quercy” of France, *C. minor* may not be an appropriate name and the large size variations attributed to this species ^76^ may imply more than one species involved ^77^. Furthermore, our knowledge about these basal ursid is still largely limited to teeth and jaws, and its cranial morphology is poorly known ^78^. We thus adopt a hypothetical ancestor approach, which is a close reflection of what *Cephalogale minor* embodies but may also include other basal cephalogalines as a composite in order to assess all cranial and dental characters. The above compromises should not affect tree topologies in the region of interest, i.e., basal Ursini.

**1. Height of lower canines** (Abella et al., 2012:char. 1). Qiu et al. (2014:char. 5) redefined the character states but their coding is similar to those of Abella et al.

Polarity: 0, canine at least twice as high as the remaining maxillary and mandibular teeth; 1, canine less than twice as high as the remaining maxillary and mandibular teeth.

**2. Relative premolar development compared to remaining teeth** ^72:char 2^.

Polarity: 0, all premolars relatively large, with developed accessory cusps; 1, morphology of the premolars less complicated, without developed accessory cusps in some of them; 2, both reduced morphology of the premolars and loss of at least one premolar.

**3. Height of coronoid process** (Abella et al., 2012:char. 3).

Polarity: 0, coronoid process does not reach twice the height of the mandibular ramus and it is relatively wide; 1, coronoid process doubles the height of the mandibular ramus and it is erect and relatively slender.

**4. Condyloid process position** (Abella et al., 2012:char. 4).

Polarity: 0, approximately the same height as the tooth row; 1, higher than the tooth row.

**5. Position of mental foramina** (Qiu et al., 2014:char. 4).

Polarity: 0, posterior-most one situated anterior to p4; 1, under p4 or more posterior.

**6. Angular process** (Abella et al., 2012:char. 6).

Polarity: 0, short, not projected caudally and with a small insertion for the *digastricus* muscle; 1, long, caudally projected and with a large insertion for the *digastricus* muscle.

**7. Insertion of *digastricus* muscle** (Abella et al., 2012:char. 9). Qiu et al. (2014:char. 3) redefined this character, but the coding by both authors are largely compatible.

Polarity: 0, lined-up with the dentary bone; 1, lingually projected, forming a curve towards the inner part of the mandible.

**8. Insertion of *zygomatico-mandibularis* muscle** (Abella et al., 2012:char. 10).

Polarity: 0, short, not covering all the rostral part of the ascending ramus; 1, long and covering the entire width of masseteric fossa in the rostral edge.

**9. Shape of ventral outline of jaw in lateral view** (Abella et al., 2012:char. 11). Qiu et al. (2014:char. 0) somewhat redefined this character but their coding is similar.

Polarity: 0, curved with most of the ventral outline of the dentary bone being convex in shape; 1, straight.

**10. Height of horizontal ramus at level of m1** (Abella et al., 2012:char. 16). Qiu et al. (2014:char. 2) redefined this character in terms of ratios of jaw depth and tooth length that are very different from that in Abella et al. The coding of these two approaches is similar.

Polarity: 0, low, not reaching thrice the height of the m1; 1, high, exceeding more than thrice the height of the m1.

**11. Mandibular symphysis ventral morphology** (Abella et al., 2012:char. 17). Qiu et al. (2014:char. 1) redefined this character but their coding seems similar to those of Abella et al.

Polarity: 0, curved; 1, marked chin.

**12. Premasseteric fossa** (Abella et al., 2012:char. 18).

Polarity: 0, absent; 1, present.

**13. Position of m3 relative to anterior rim of coronoid process** (Abella et al., 2012:char. 19).

Polarity: 0, in front of the coronoid process, well-separated from the ascending ramus; 1, partial overlapping with coronoid process, touching the front edge of ascending ramus; 2, behind coronoid process, partly or completely covered by the coronoid process in lateral view.

**14. P1 (p1) enlargement** (Qiu et al., 2014:char. 6).

Polarity: 0, small relative to other ante-carnassial premolars; 1, larger than other ante-carnassial premolars.

**15. P1-P3 and p1-p4 diastema** (Abella et al., 2012:char. 22).

Polarity: 0, absent; 1, present.

**16. Loss of p2** (Abella et al., 2012:char. 23).

Polarity: 0, present; 1, absent.

**17. Loss of p3** (Abella et al., 2012:char. 24).

Polarity: 0, present; 1, absent.

**18. Posterior accessory cuspid of p4** (Abella et al., 2012:char. 25). Qiu et al. (2014:char. 12) defined this character differently and coded it somewhat differently as well. We follow Abella et al.

Polarity: 0, present; 1, absent.

**19. m1 paraconid and protoconid** (Abella et al., 2012:char. 27).

Polarity: 0, sharp and blade-like; 1, bunodont and cusp-like.

**20. Height of m1 protoconid relative to paraconid** (Abella et al., 2012:char. 29).

Polarity: 0, protoconid clearly higher than the paraconid; 1, protoconid equal or subequal in height to paraconid.1

**21. Size and position of m1 metaconid** (Abella et al., 2012:char. 31). Qiu et al. (2014:char. 31) redefined this character to highlight developments in the Ailuropodinae clade and consequently coded it very differently from that of Abella et al. We combine both approaches and add an additional state.

Polarity: 0, modest size and positioned on the lingual side of protoconid; 1, reduced and positioned on the posterolingual aspect of protoconid; 2, enlarged, subequal to height of paraconid.

**22. m1 pre-metaconid** (Abella et al., 2012:char. 32). Abella et al. coded a presence of this small cusp in *Melursus ursinus* and its absence in *Helarctos malayanus*, observations that are contrary to that by Erdbrink ^6^. We follow the latter author in our coding see further discussion in ^7^.

Polarity: 0, absent; 1, present.

**23. Labial part of m1 talonid** (Abella et al., 2012:char. 33).

Polarity: 0, narrow, without expansion; 1, widened, creating an expansion.

**24. Lingual ridge on m1 protoconid** (Abella et al., 2012:char. 35).

Polarity: 0, absent; 1, present.

**25. Posterior wall of m1 trigonid in lingual view** (Abella et al., 2012:char. 37). Qiu et al. (2014:char. 34) gave a more quantitative definition of this character, which is followed here.

Polarity: 0, subvertical, usually more than 60° with horizontal line; 1, anteriorly inclined, forming an angle around 45 with horizontal.

**26. m1 trigonid posterior face** (Erdbrink, 1953). This is also known as “V-, Y-, X-shaped ridges” by Qiu et al. (2009).

Polarity: 0, posterior surface without zigzag pattern; 1, with zigzag pattern,.

**27. Length of m1 talonid relative to trigonid** (Abella et al., 2012:char. 38). Qiu et al. (2014:char. 28) gave a more quantitative definition but their coding is identical to that of Abella et al.

Polarity: 0, short; 1, long.

**28. m1 pre-hypoconid** (Abella et al., 2012:char. 39). Qiu et al. (2014:char. 30) adopted this character but have somewhat different coding in their matrix.

Polarity: 0, absent; 1, present.

**29. m1 entoconid** (Qiu et al., 2014:char. 32). Certain taxa are known to have both conditions, such as in *Helarctos malayanus* ^6:plates V-VIII^.

Polarity: 0, single cusplet or crested; 1 double cusps.

**30. m1 hypoconulid ridge** (Abella et al., 2012:char. 45).

Polarity: 0, absent or poorly-developed, not closing the talonid valley posteriorly; 1, well-developed, closing the talonid valley between entoconid and hypoconid.

**31. m2 length relative to that of m1** (Abella et al., 2012:char. 46). Qiu et al. (2014:char. 35) gave a somewhat more quantitative definition about the character states but their coding is identical to that of Abella et al.

Polarity: 0, relatively short, less than half the length of m1; 1, relatively long, typically more than half the length of the m1 and can be greater than 0.7–0.8 times of m1 length or more.

**32. Size of m2 paraconid in relation to rest of cuspids in trigonid** (Abella et al., 2012:char. 47).

Polarity: 0, small or absent; 1, large, sub-equal in size to protoconid.

**33. m2 pre-metaconid** (Abella et al., 2012:char. 48).

Polarity: 0, absent; 1, present.

**34. Relative size of m2 talonid compared to trigonid** (Abella et al., 2012:char. 49).

Polarity: 0, shorter than trigonid; 1, longer than trigonid.

**35. Relative size of m2 protoconid and metaconid** (Abella et al., 2012:char. 50).

Polarity: 0, protoconid taller than metaconid; 1, metaconid subequal or taller than protoconid.

**36. m2 pre-entoconid (entoconulid)** (Abella et al., 2012:char. 52).

Polarity: 0, absent; 1, present.

**37. m2 labial cingulum relative to remaining cingulids** (Abella et al., 2012:char. 53).

Polarity: 0, small, not surrounding the whole labial wall of m2; 1, large, running the entire labial wall of m2.

**38. m3 size relative to m2** (Abella et al., 2012:char. 54). Qiu et al. (2014:char. 36) gave a higher cut-off point for relative size of m3 compared to Abella et al.’s definition, thereby highlighting the conditions in more derived taxa in advanced species of *Ursavus* and *Ailuractos*. We attempt to combine both approaches and add a new state.

Polarity 0, small, less than 1/4 the length of m2; 1, large, at least 1/4 but less than 2/3 of the length of m2; 2, larger, 2/3 or more of m2.

**39. m3 morphology in occlusal view** (Abella et al., 2012:char. 55).

Polarity: 0, rounded or ovoid; 1, subtriangular with pointed tip usually on posterior end.

**40. Reduction of P4 blade length relative to M1** (Abella et al., 2012:char. 58). Qiu et al. (2014:char16) redefined this as a 3-state character, in contrast to a 2-state character by Abella et al. We follow Qiu et al.

Polarity: 0, carnassial blade (including parastyle, if present) longer than M1; 1, about equally long or slightly shorter than M1; 2, markedly shorter than M1.

**41. P4 parastyle** (Abella et al., 2012:char. 59). Qiu et al. (2014:char. 15) redefined the character states and coded *Kretzoiarctos* differently from Abella et al. We follow the coding of Abella et al.

Polarity: 0, absent; 1, present but small; 2, present and well-developed, distinct from the paracone.

**42. P4 protocone relative size in occlusal view** (Abella et al., 2012:char. 60).

Polarity: 0, small, much smaller than remaining cusps in trigon; 1, large, similar in size to paracone and metastyle.

**43. P4 protocone** (Abella et al., 2012:char. 61). Qiu et al. (2014:char. 17) split this character into three states in contrast to two states by Abella et al. The additional state is mostly developed in the panda clade, which is not very relevant in this study, and we follow Abella in the more simplified coding.

Polarity: 0, simple, only one cusp; 1, complex, with more than one cusp and/or surrounded by a cingulum.

**44. P4 protocone position** (Abella et al., 2012:char. 62). Qiu et al. (2014:char. 18) added an additional state to this character to further delineate stage of evolution in more advanced ursines. We follow that of Qiu et al.

Polarity: 0, anterior to paracone, extending anterolingually; 1, opposite paracone, extending lingually; 2, posterior to paracone with its posterior end reaching to or passing beyond the carnassial notch.

**45. M1 metaconule** (Abella et al., 2012:char. 65). Abella et al. (2012) called this cusp a hypocone, but in most ursids the cusp at the posterolingual corner of the M1 is actually homologous to metaconule see ^79:fig. 1^.

Polarity: 0, absent; 1, present.

**46. M1 crown shape** (Abella et al., 2012:char. 66). Qiu et al. (2014:char. 20) redefined and added one state in this character, which is followed in this study.

Polarity: 0, trapezoid, wider than long, with lingual border much shorter than labial border; 1, subquadrate, with convex lingual border; 2, longer than wide, lingual side more or less straight.

**47. M1 postprotocrista** (Abella et al. 2012:char. 67+68). Qiu et al. (2014:char. 22) combined two of Abella et al.’s characters, which is followed in this study.

Polarity: 0, present; 1, absent.

**48. M1 lingual cusps** (Qiu et al., 2014:char. 23).

Polarity: 0, protocone V-shaped, metaconule situated posterolabial to protocone; 1, protocone and metaconule situated in the same longitudinal line, forming a very shallow W-shaped crest; 2, protocone, postprotocrista and metaconule forming a longitudinal crest.

**49. M1 lingual cingulum** (Abella et al. 2012:char. 69). Qiu et al. (2014:char. 24) redefined and added one state for this character, which is followed here.

Polarity: 0, confluent with anterior cingulum, forming a strong curve and deep groove lingual to lingual cusps; 1, shelf- or ridge-like, extending from anterior end of lingual side to metaconule apex; 2, strongly attenuated, may not be continuous.

**50. M2 length relative to M1** (Abella et al. 2012:char. 70). Qiu et al. (2014:char. 25) redefined this character and added one state. We further divide it into four states.

Polarity: 0, shorter than M1; 1, subequal to M1; 2, longer than M1 but less than 1.5 times as long; 3, more than 1.5 times as long as M1.

**51. M2 talon** (Abella et al. 2012:char. 71). Qiu et al. (2014:char. 26) redefined this character. We follow Abella et al.’s usage.

Polarity: 0, absent or small; 1, present but moderately-development, not being longer than the trigon; 2, present and well-developed, longer than trigon.

**52. Enamel surface on molars** (Abella et al., 2012:char. 73).

Polarity: 0, enamel surface smooth; 1, wrinkled surface.

**53. Rostral maximum width of skull** (Abella et al., 2012:char. 75).

Polarity: 0, attained at the level of P4; 1, attained at the level of upper canines.

**54. Sagittal crest in lateral view** (Abella et al., 2012:char. 76).

Polarity: 0, caudally projected towards the cervical vertebra; 1, obliquely-inclined and moderately-developed, but not surpassing the level of the nuchal crest; 2, dorsally-projected, with its highest level not situated in the dorsal-most part of the crest.

**55. Mastoid process** (Abella et al., 2012:char. 77).

Polarity: 0, small; 1, well-developed, protruding laterally and ventrally passing the lower level of zygomatic arch.

**56. Paraoccipital process** (Abella et al., 2012:char. 78).

Polarity: 0, short, not surpassing the ventral level of the mastoid process; 1, long and ventrally projected, longer or subequal to the mastoid process.

**57. Posterior margin of palatine in ventral view** (Abella et al., 2012:char. 79).

Polarity: 0, at about the level of posterior edge of M2; 1, modestly expanded posteriorly beyond the posterior edge of M2; 2, further expanded and separated from M2 by at least one

length of the M2.

**58. Alisphenoid canal** (Abella et al., 2012:char. 80). Abella et al. (2012) coded a loss of alisphenoid canal in all living ursids, which is not the case – only the giant panda has lost this canal ^80^.

Polarity: 0, present; 1, absent.

**59. Zygomatic arch in lateral view** (Abella et al., 2012:char. 81). We have coded this differently from that by Abella et al. (2012).

Polarity: 0, narrow, with central portion never being wider than its rostral or caudal parts; 1, wide, with central portion wider than its rostral and caudal parts.

**Table S6.** Data matrix for phylogenetic analysis (Fig. 7). This 24 taxa by 59 character matrix combines elements of Abella et al. (2012) and Qiu et al. (2014), the only cladistic analyses of fossil ursids. Table S1 describes individual characters and how we differ from previous authors. Numbers in “[]” indicate multi-state coding.

Hypothetical ancestor 00000000000000000000000000000000000000000000000000000000000

*Zaragocyon daamsi* 01????????????00000010000010?11011100??0010110010001???????

*Ballusia elmensis* 10??0?0?000000000000100?1010101?111?01?1000111011001???????

*Ballusia orientalis* ??????0?00???0??????1???10101?1??????1?20?01?111100????????

*Ursavus brevirhinus* 10??0?0?010??00001001011101011101111?101000112121111???????

*Indarctos vireti* 11?1?11101001?00011110011011?110111111010101111211110?11001

*Indarctos arctoides* 1111?11101001?00011110111011?110111111011111111211110111001

*Indarctos punjabiensis* 1211?11101001?00011110111011?110111111012111111211110?10001

*Kretzoiarctos beatrix* 11????1?010010000011100?1010111011111101211111121??1???????

*Agriarctos depereti* ?0??????????????0011200?10101110111111012111?11211?1???????

*Ailurarctos lufengensis* ?0???????????????011201?10111?1011111211211212121221???????

*Ailuropoda melanoleuca* 10111111010020000011201110110010111112012112121212210210011

*Ursavus primaevus* ????1?1?000???????0010011010111??11??101000112121211???????

*Ursavus tedfordi* 1100111111101100010010001010101011110102000211122211101?100

*Protarctos abstrusus* ?1??1?1110001100011110011010111011?101?20002121212211011?00

*Protarctos yinanensis* 01?011?1100001000111100110100010111101020002121212211???1?0

*Euarctos minimus* 0?00????10000????111110011100[0 1]1011110??20002121212111???2??

*Tremarctos ornatus* 11001111100111000111101010110111111102120102121213211110100

*Melursus ursinus* 01001111101000000111101010100011111001020102121212111111200

*Helarctos malayanus* 020011111100110101111[0 1]001010[0 1]110111101020002121212111010200

*Ursus thibetanus* 02001111100011010111110011100110111101[0 1]20002121212111011200

*Ursus americanus* 02001111100011110111110011100010111102020002121213211010200

*Ursus arctos* 02001111101000111111110010110010111102120102121213211010201

*Ursus maritimus* 02001111101000111011110010101110111102120002121213211010201

**
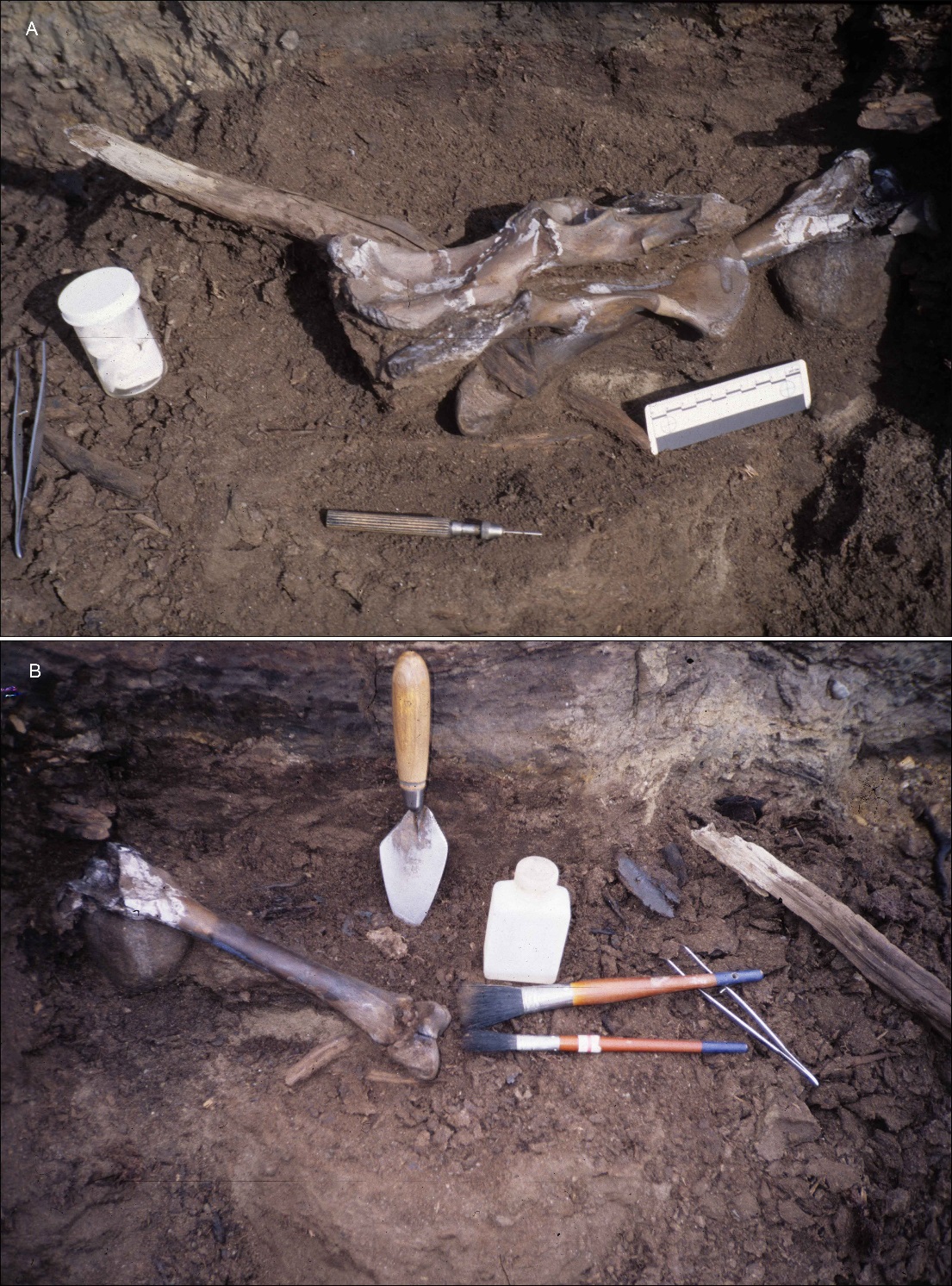
**

**Figure S1**. Beaver Pond site excavations. A, nearly complete right and left pelves of *Protarctos abstrusus* (CMN 51779-A) as excavated in place on July 19, 1997, showing them compressed together with proximal end of left femur (CMN 51779-B) below and to the right. A large beaver-cut stick lies between the innominates, its tip projecting left of the pelves. B, nearly complete left femur [which articulates with the left pelvis (removed)], as exposed on July 20, 1997, showing excavation tools (photos by C.R. Harington).


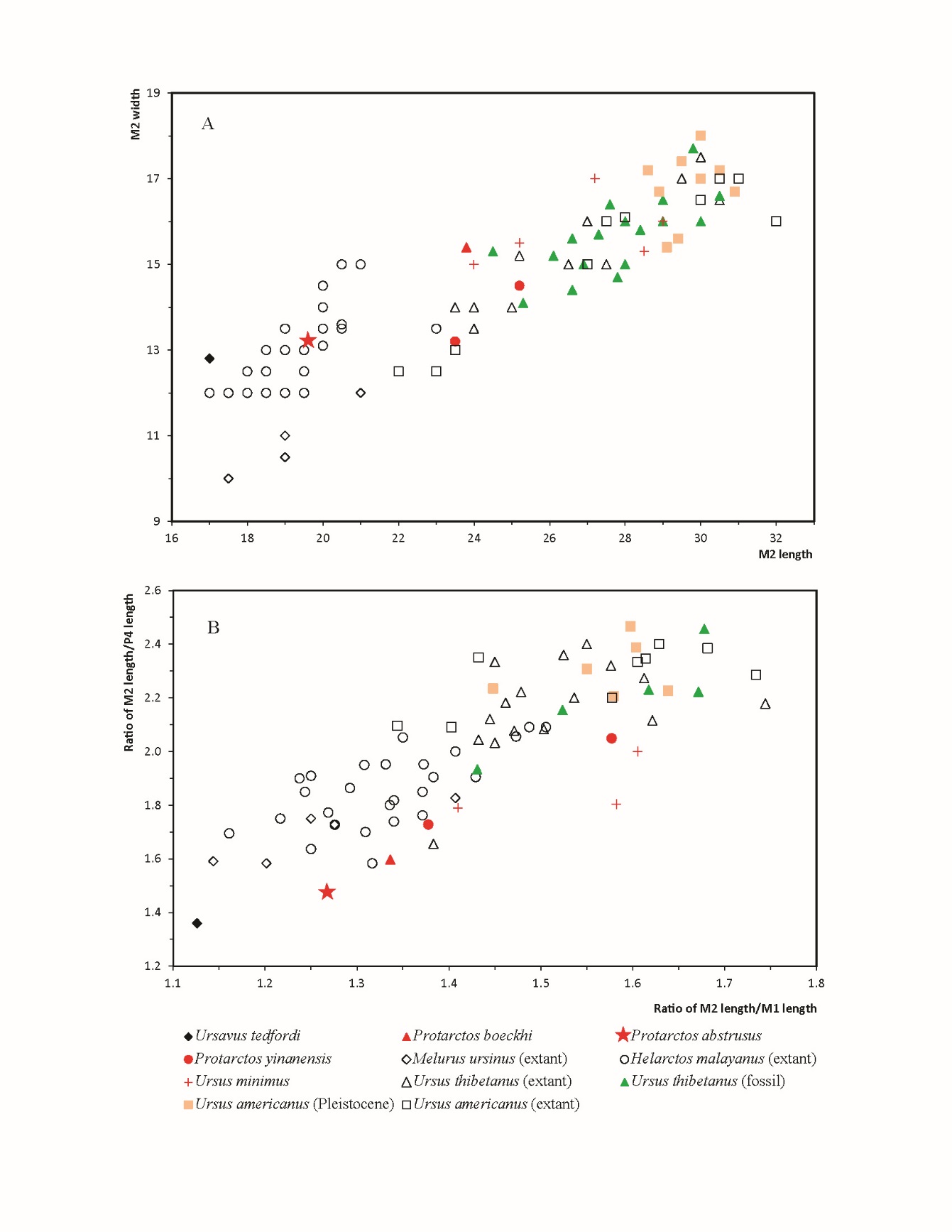


**Figure S2**. Upper molar proportions in ursines. A, plots of M2 length vs. M2 width for fossil and living ursines showing trends in M2 elongation; B, plots of ratios of M2 length/M1 length vs M2 length/P4 length showing proportional lengthening of posterior molars at the expense of upper carnassial. Measurements of living ursines were mostly compiled by Erdbrink (1953:tables I-VII) and those for extinct ursines were from various authors (Supplementary Table S2).


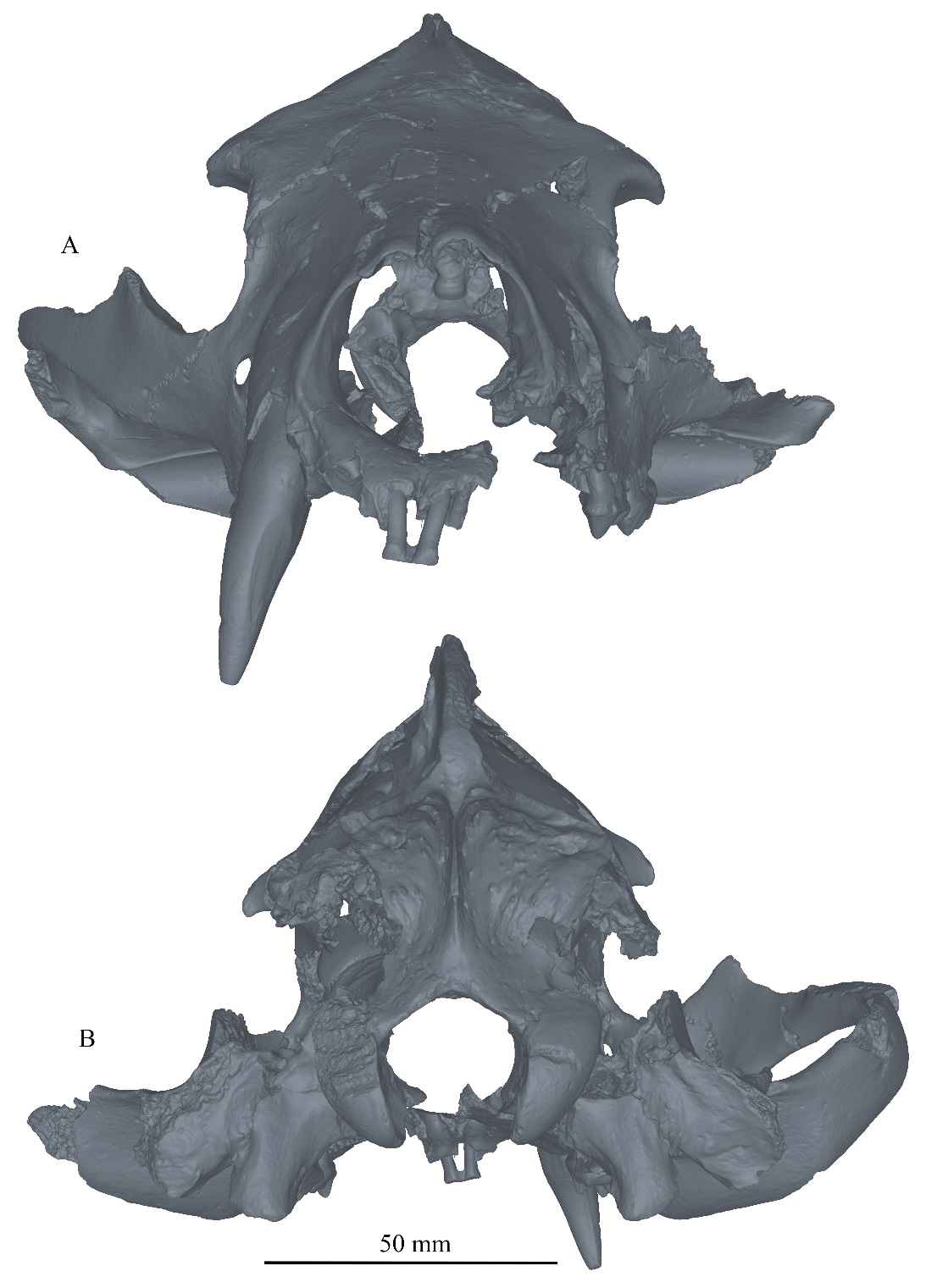


**Figure S3.** Anterior (A) and posterior (B) views of skull of *Protarctos abstrusus*. composite laser scans of five individual cranial fragments as assembled in Avizo Lite (version 9.0.0) and visualized in PointStream 3D Image Suite (Version 3.2.0.0).

**
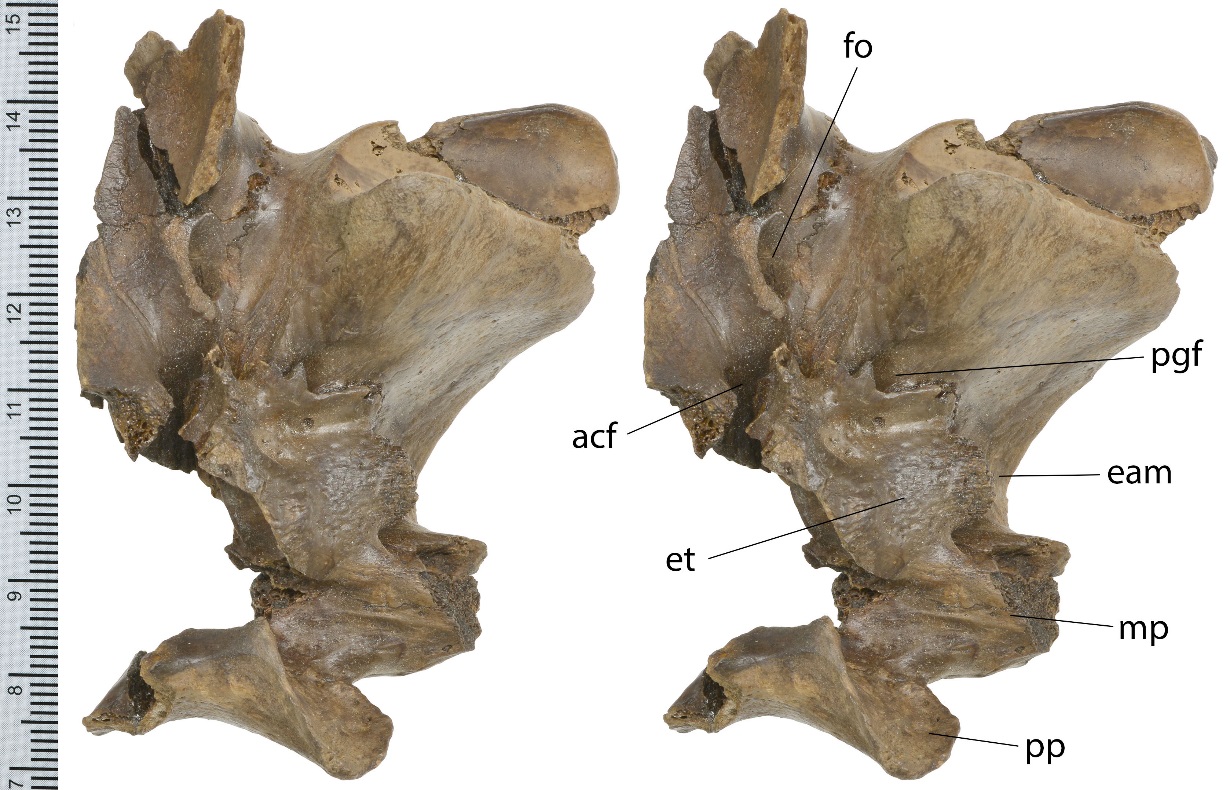
**

**Figure S4**. Stereo photos of left basicranial region of *Protarctos abstrusus*, CMN 54380, ventral view. Abbreviations: acf, anterior carotid foramen; eam, external auditory meatus; et, ectotympanic; fo, foramen ovale; mp, mastoid process; pgf, postglenoid foramen; pp, paroccipital process.


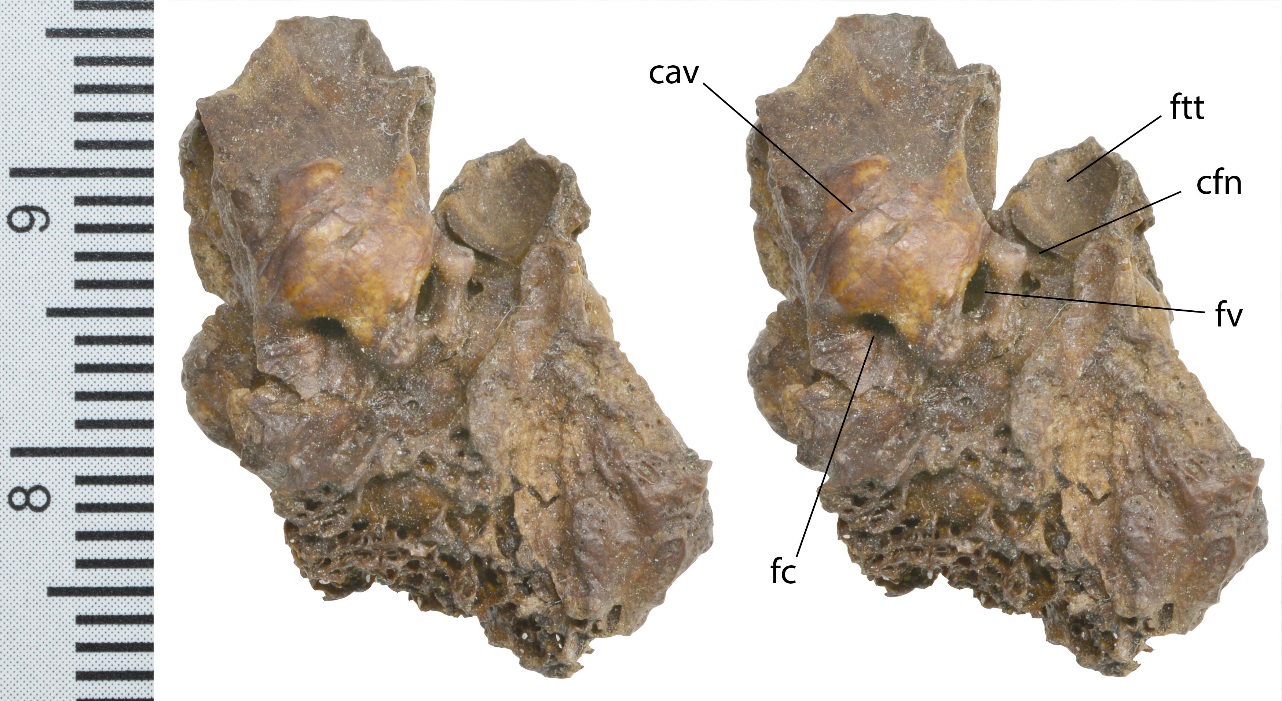


**Figure S5**. Stereo photos of left petrosal of *Protarctos abstrusus*, CMN 54380, ventral view. Abbreviations: cav, caroticotymanic arteries and nerves; cfn, canal for facial nerve; fc, fenestra cochleae (fenestra rotunda); fv, fenestra vestibuli (fenestra ovalis); ftt, fossa for tensor tympani muscle.


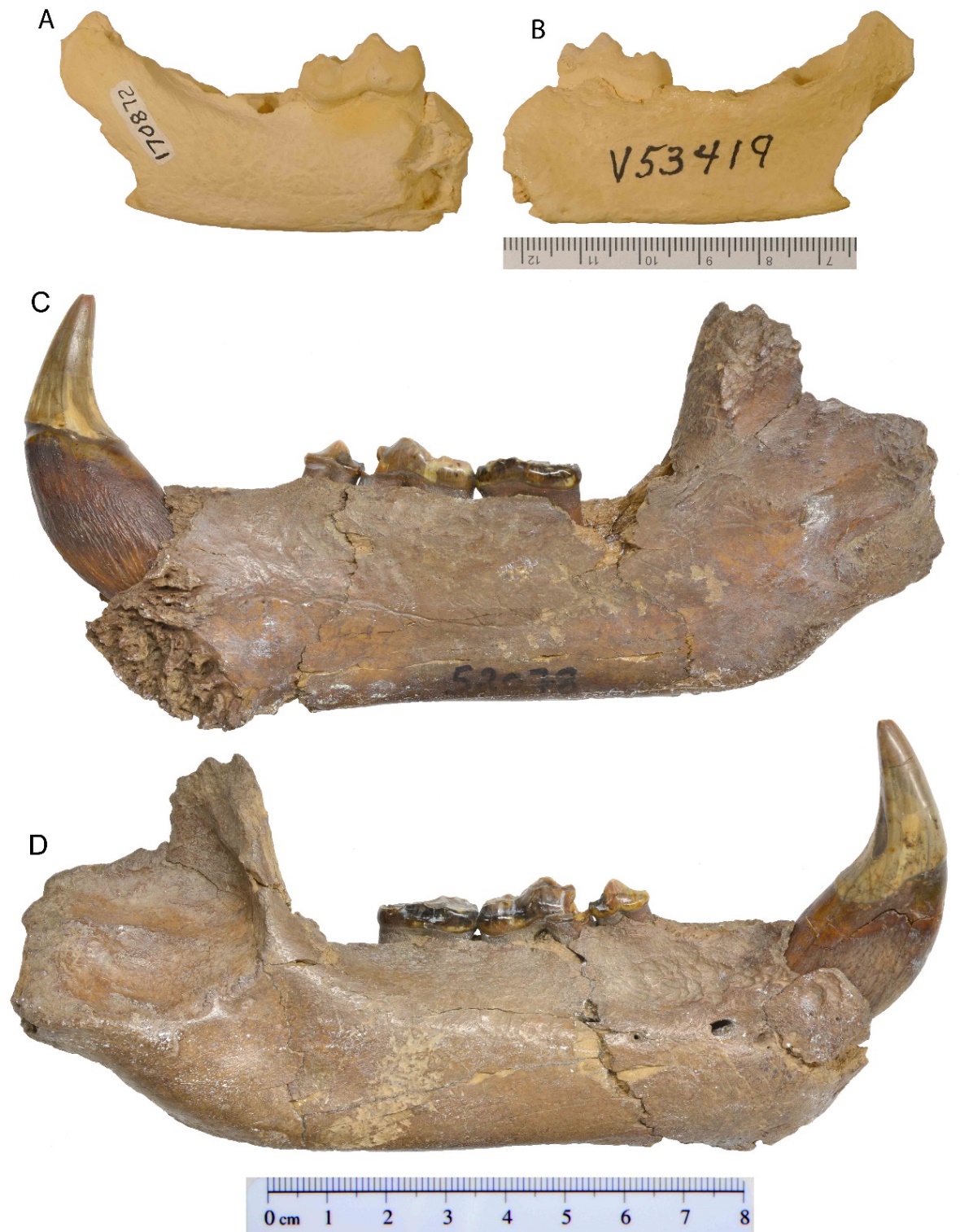


**Figure S6**. Right dentaries of *Protarctos abstrusus*, A, lingual, and B, buccal views of holotype, a cast of UMMP V53419 from the National Museum of Natural History (USNM 170872); C, lingual, and D, buccal views of CMN 52078B, Beaver Pond Site.

**Supplementary Information References**

1 Hunt, R. M., Jr. The auditory bulla in Carnivora: an anatomical basis for reappraisal of carnivore evolution. *J. Morphol.* **143**, 21-76 (1974).

2 Story, H. E. The carotid arteries in the Procyonidae. *Fieldiana Zool.* **32**, 477-557 (1951).

3 Qiu, Z.-x., Deng, T. & Wang, B.-y. A Late Miocene *Ursavus* skull from Guanghe, Gansu, China. *Vert. PalAsiat.* **52**, 265-302 (2014).

4 Ryziewicz, Z. Studies on Pliocene bears. *Acta Palaeontol. Polonica* **14**, 199-243 (1969).

5 Wagner, J. & Čermák, S. Revision of the early Middle Pleistocene bears (Ursidae, Mammalia) of Central Europe, with special respect to possible co-occurrence of spelaeoid and arctoid lineages. *Bulletin of Geosciences* **87**, 461-496 (2012).

6 Erdbrink, D. P. *A review of fossil and recent bears of the Old World with remarks on their phylogeny based upon their dentition. Part I*. (Drukkerij Jan de Lange, 1953).

7 Qiu, Z.-x., Deng, T. & Wang, B.-y. First ursine bear material from Dongxiang, Gansu -- addition to the Longdan Mammalian Fauna (2). *Vert. PalAsiat.* **47**, 245-264 (2009).

8 Bjork, P. R. The Carnivora of the Hagerman local fauna (late Pliocene) of southwestern Idaho. *Transactions of the American Philosophical Society, Philadelphia, New Series* **60**, 1-54 (1970).

9 Ruez, D. R., Jr. *Effects of climate change on mammalian fauna composition and structure during the advent of North American continental glaciation in the Pliocene* Ph.D. thesis, University of Texas at Austin, (2007).

10 Ruez, D. R., Jr. Revision of the Blancan (Pliocene) mammals from Hagerman Fossil Beds National Monument, Idaho. *Journal of the Idaho Academy of Science* **45**, 1-143 (2009).

11 Bell, C. J. *et al.* in *Late Cretaceous and Cenozoic mammals of North America: Biostratigraphy and Geochronology* (ed Michael O. Woodburne) 232-314 (Columbia University Press, 2004).

12 Repenning, C. A. in *Cenozoic mammals of North America, geochronology and biostratigraphy* (ed Michael O. Woodburne) 236-268 (University California Press, 1987).

13 Rybczynski, N. *et al.* Mid-Pliocene warm-period deposits in the High Arctic yield insight into camel evolution. *Nat. Commun.* **4**, 1550, doi:<http://www.nature.com/ncomms/journal/v4/n3/suppinfo/ncomms2516_S1.html> (2013).

14 Gustafson, E. P. The vertebrate faunas of the Pliocene Ringold Formation, south-central Washington. *Bull. Mus. Nat. Hist. Univ. Oregon* **23**, 1-62 (1978).

15 Tedford, R. H. & Martin, J. *Plionarctos*, a tremarctine bear (Ursidae; Carnivora) from western North America. *J. Vert. Paleontol.* **21**, 311-321 (2001).

16 Johnston, C. S. & Savage, D. E. A survey of various late Cenozoic vertebrate faunas of the Panhandle of Texas, Part I: Introduction, description of localities, preliminary faunal lists. *University of California Publications of Geological Science* **31**, 27-50 (1955).

17 Kurtén, B. Notes on some Pleistocene mammal migrations from the Palearctic to the Nearctic. *Eiszeitalter und Gegenwart* **14**, 96-103 (1963).

18 Hunt, R. M., Jr. in *Evolution of Tertiary Mammals of North America, Volume 1: Terrestrial Carnivores, Ungulates, and Ungulatelike Mammals* (eds Christine M. Janis, Kathleen M. Scott, & Louis L. Jacobs) 174-195 (Cambridge University Press, 1998).

19 Harington, C. R. Life at a 3.5 million-year-old beaver pond in the Canadian Arctic Islands and the modern scene. *Meridian* **Fall/Winter**, 11-13 (2001).

20 Harington, C. R. Life at an early Pliocene beaver pond in the Canadian high arctic. *J. Vert. Paleontol.* **23**, 59A (2003).

21 Tedford, R. H. & Harington, C. R. An Arctic mammal fauna from the Early Pliocene of North America. *Nature* **425**, 388-390 (2003).

22 Schlosser, M. *Parailurus anglicus* und *Ursus böckhi*, aus den Ligniten von Baróth-Köpecz, Comitat Háromzèk in Ungarn. *Mittheilungen aus dem Jahrbuche der Königlichen Ungarischen Geologischen Anstalt* **13**, 66-95 (1899).

23 Maier von Mayerfels, S. Zur Stammesgeschichte der europäischen Bären. *Neues Jahrbuch für Mineralogie, Geologie und Paläontologie* **62**, 325-332 (1929).

24 Kretzoi, M. Bemerkungen über das Raubtiersystem. *Annales Historico-Naturales Musei Nationalis Hungarici* **38**, 59-83 (1945).

25 Li, Y.-z. On a new species of Pliocene *Ursus* (Carnivora: Ursidae) from Yinan, Shandong Province. *Vert. PalAsiat.* **31**, 44-60 (1993).

26 Morlo, M. & Kundrát, M. The first carnivoran fauna from the Ruscinium (Early Pliocene, MN 15) of Germany. *Paläont. Z.* **75**, 163-187 (2001).

27 Wagner, J., Čermák, S. & Horáček, I. The presence of *Ursus* ex gr. *minimus-thibetanus* in the late Villányian and its position among the Pliocene and Pleistocene black bears in Europe. *Quaternaire* **4**, 39-58 (2011).

28 Mazza, P. & Rustioni, M. On the phylogeny of Eurasian bears. *Palaeontographica Abteilung A* **230**, 1-38 (1994).

29 Depéret, C. Les animaux pliocène du Rousillon. *Mémoires de la Société Géologique de France, Paléontologie* **3**, 1-164 (1890).

30 Devèze de Chabriol, J. S. & Bouillet, J.-B. *Essai géologique et minéralogique sur les environs d'Issoire, département du Puy-de-Dôme, et principalement sur la Montagne de Boulade, avec la description et les figures lithographiées des ossemens fossiles qui y ont été recueillis*. (Imprimerie de Thibaud-Landriot, 1827).

31 Croizet, l. A. & Jobert, A. C. G. *Recherches sur les ossemens fossiles du départment du Puy-de-Dôme*. (Delahays, 1828).

32 Kurtén, B. *Pleistocene Mammals of Europe*. 317 (Aldine Publishing Company, 1968).

33 Wagner, J., Lipecki, G. & Krawczyk, M. New evidence of *Ursus minimus* from the territory of Poland. *Stalactite* **58**, 78-80 (2008).

34 Baryshnikov, G. F. & Zakharov, D. S. Early Pliocene bear *Ursus thibetanus* (Mammalia, Carnivora) from Priozernoe locality in the Dniester Basin (Moldova Republic). *Proceedings of the Zoological Institute of Russian Academy of Sciences* **317**, 3-10 (2013).

35 Vangengeim, E. A., Vislobokova, I. & Sotnikova, M. V. Large Ruscinian Mammalia in the territory of the former Soviet Union. *Stratigraphy and Geological Correlation* **6**, 52-66 (1998).

36 Wagner, J. A list of craniodental material of Pliocene ursids (genus *Ursus*) in the collection of Naturhistorisches Museum Basel. *Scientific Annals School of Geology Aristotle University of Thessaloniki* **98**, 127-139 (2006).

37 Wagner, J. Pliocene to early Middle Pleistocene ursine bears in Europe: a taxonomic overview. *Journal of the National Museum (Prague), Natural History Series* **179**, 197-215 (2010).

38 Sotnikova, M. V. in *International Symposium on Stratigraphy, Paleontology and Paleoenvironment of Pliocene-Pleistocene of Transbaikalia and Interregional Correlations* (eds Nadezhda V. Alexeeva, Margarita A. Erbajeva, & A. G. Mironov) 84-85 (INQUA Commission on Stratigraphy and Chronology, 2006).

39 Erbajeva, M. A. & Alexeeva, N. V. in *Fossil Mammals of Asia: Neogene Biostratigraphy and Chronology* (eds Xiaoming Wang, Lawrence J. Flynn, & Mikael Fortelius) 495-507 (Columbia University Press, 2013).

40 Zheng, S.-h. A new species of *Kowalskia* (Rodentia, Mammalia) of Yinan, Shandong. *Vert. PalAsiat.* **22**, 251-260 (1984).

41 Sabol, M., Holec, P. & Wagner, J. Late Pliocene carnivores from Včeláre 2 (Southeastern Slovakia). *Paleontological Journal* **42**, 531-543 (2008).

42 Baryshnikov, G. F. & Lavrov, A. V. Pliocene bear *Ursus minimus* Devèze de Chabriol et Bouillet, 1827 (Carnivora, Ursidae) in Russia and Kazakhstan. *Russian Journal of Theriology* **12**, 107-118 (2013).

43 Qiu, Z.-x., Deng, T. & Wang, B. Y. Early Pleistocene mammalian fauna from Longdan, Dongxiang, Gansu, China. *Palaeontol. Sinica N. S. C* **27**, 1-198 (2004).

44 Heller, F. *Ursus* (*Plionarctos*) *stehlini* Kretzoi, der kleine Bär aus den altdiluvialen Sanden von Mauer-Bammerntal und Mainz-Wiesbaden. *Beiträge zur Geologie und Paläontologie des Tertiäres und des Diluviums in der Umgebung von Heidelberg, Bitzungsberichte der Heidelberger Akademie der Wissenshaften, Matematisch-Naturwissenschaftliche Klasse* **11**, 451-508 (1949).

45 Teilhard de Chardin, P. The fossils from Locality 18 near Peking. *Palaeontologia Sinica (new series C)* **9**, 1-101 (1940).

46 Zdansky, O. Weitere bemerkungen über fossile carnivoren aus China. *Palaeontol. Sinica S. C* **4**, 1-28 (1927).

47 Thenius, E. Remerkungen über fossile Ursiden (Mamm.). *Sitzungsberichte der Österreichische Akademie der Wissenschaften in Wien Mathematisch-Naturwissenschafttliche Klasse, Abteilung I* **156**, 201-208 (1947).

48 Teilhard de Chardin, P. & Piveteau, J. Les mammifères fossiles de Nihowan (Chine). *Ann. Paléontol.* **19**, 1-134 (1930).

49 Zdansky, O. Die Säugetiere der Quartärfauna von Chou-K'ou-Tien. *Palaeontol. Sinica Ser. C* **5**, 1-146 (1928).

50 Pei, W.-c. On the Carnivora from Locality 1 of Choukoutien. *Palaeontologia Sinica (series C)* **8**, 1-216 (1934).

51 Matthew, W. D. & Granger, W. New fossil mammals from the Pliocene of Sze-Chuan, China. *Bull. Am. Mus. Nat. Hist.* **48**, 563-598 (1923).

52 Liu, J.-y. & Qiu, Z.-x. in *Paleolithic Site -- the Renzidong Cave, Fanchang, Anhui Province* (eds Chang-zhu Jin & Jin-yi Liu) 220-283 (Science Press, 2009).

53 Jiangzuo, Q.-g., Cong, H.-l., Ma, R., Feng, H. & Liu, J.-y. in *Proceedings of the Fourteenth Annual Meeting of the Chinese Society of Vertebrate Paleontology* Vol. 2014 (ed Wei Dong) 119-134 (China Ocean Press, 2014).

54 Cronin, M. A. *et al.* Molecular phylogeny and SNP variation of polar bears (*Ursus maritimus*), brown bears (*U. arctos*), and black bears (*U. americanus*) derived from genome sequences. *J. Heredity* **105**, 312-323, doi:10.1093/jhered/est133 (2014).

55 Miller, W. *et al.* Polar and brown bear genomes reveal ancient admixture and demographic footprints of past climate change. *Proc. Nat. Acad. Sci.* **109**, E2382–E2390, doi:10.1073/pnas.1210506109 (2012).

56 Hailer, F. *et al.* Nuclear genomic sequences reveal that polar bears are an old and distinct bear lineage. *Science* **336**, 344-347, doi:10.1126/science.1216424 (2012).

57 Cronin, M. A. & MacNeil, M. D. Genetic relationships of extant brown bears (*Ursus arctos*) and polar bears (*Ursus maritimus*). *J. Heredity* **103**, 873-881, doi:10.1093/jhered/ess090 (2012).

58 Lindqvist, C. *et al.* Complete mitochondrial genome of a Pleistocene jawbone unveils the origin of polar bear. *Proc. Nat. Acad. Sci.* **107**, 5053-5057, doi:10.1073/pnas.0914266107 (2010).

59 Agnarsson, I., Kuntner, M. & May-Collado, L. J. Dogs, cats, and kin: A molecular species-level phylogeny of Carnivora. *Mol. Phyl. Evol.* **54**, 726-745, doi:<http://dx.doi.org/10.1016/j.ympev.2009.10.033> (2010).

60 Krause, J. *et al.* Mitochondrial genomes reveal an explosive radiation of extinct and extant bears near the Miocene-Pliocene boundary. *BMC Evol. Biol.* **8**, 220 (2008).

61 Bon, C. *et al.* Deciphering the complete mitochondrial genome and phylogeny of the extinct cave bear in the Paleolithic painted cave of Chauvet. *Proc. Nat. Acad. Sci.*, doi:10.1073/pnas.0806143105 (2008).

62 Fulton, T. L. & Strobeck, C. Molecular phylogeny of the Arctoidea (Carnivora): Effect of missing data on supertree and supermatrix analyses of multiple gene data sets. *Mol. Phyl. Evol.* **41**, 165-181 (2006).

63 Yu, L., Li, Q.-w., Ryder, O. A. & Zhang, Y.-p. Phylogeny of the bears (Ursidae) based on nuclear and mitochondrial genes. *Mol. Phyl. Evol.* **32**, 480-494 (2004).

64 Pagès, M. *et al.* Combined analysis of fourteen nuclear genes refines the Ursidae phylogeny. *Mol. Phyl. Evol.* **47**, 73-83, doi:<http://dx.doi.org/10.1016/j.ympev.2007.10.019> (2008).

65 Nakagome, S., Pecon-Slattery, J. & Masuda, R. Unequal rates of Y chromosome gene divergence during speciation of the family Ursidae. *Mol. Biol. Evol.* **25**, 1344-1356, doi:10.1093/molbev/msn086 (2008).

66 Kutschera, V. E. *et al.* Bears in a forest of gene trees: Phylogenetic inference is complicated by incomplete lineage sorting and gene flow. *Mol. Biol. Evol.* **31**, 2004–2017, doi:10.1093/molbev/msu186 (2014).

67 Zhang, Y.-p. & Ryder, O. A. Phylogenetic relationships of bears (the Ursidae) inferred from mitochondrial DNA sequences. *Mol. Phyl. Evol.* **3**, 351 - 359 (1994).

68 Talbot, S. L. & Shields, G. F. A phylogeny of the bears (Ursidae) inferred from complete sequences of three mitochondrial genes. *Mol. Phyl. Evol.* **5**, 567-575 (1996).

69 Yu, L., Li, Y.-W., Ryder, O. & Zhang, Y.-P. Analysis of complete mitochondrial genome sequences increases phylogenetic resolution of bears (Ursidae), a mammalian family that experienced rapid speciation. *BMC Evol. Biol.* **7**, 198 (2007).

70 Kurtén, B. Fossil bears from Texas. *Pearce-Sellards Ser Texas Mem Mus.* **1**, 1-15 (1963).

71 Kumar, V. *et al.* The evolutionary history of bears is characterized by gene flow across species. *Sci Rep* **7**, 46487, doi:10.1038/srep46487 (2017).

72 Abella, J. *et al.* *Kretzoiarctos* gen. nov., the oldest member of the giant panda clade. *PLoS ONE* **7**, e48985, doi:10.1371/journal.pone.0048985 (2012).

73 Hall, E. R. Supernumerary and missing teeth in wild mammals of the order Insectivora and Carnivora, with some notes on disease. *Journal of Dental Research* **19**, 103-143 (1940).

74 Manville, A. M. I. in *Bears: Their Biology and Management* Vol. 8 *A Selection of Papers from the Eighth International Conference on Bear Research and Management* 129-134 (International Association for Bear Research and Management, 1989).

75 Marks, S. A. & Erickson, A. W. Age determination in the black bear. *The Journal of Wildlife Management* **30**, 389-410 (1966).

76 Beaumont, G. d. Contribution à l'Étude du genre *Cephalogale* Jourdan (Carnivora). *Schweiz. Paläont. Abh.* **82**, 1-34 (1965).

77 Bonis, L. d. Ursidae (Mammalia, Carnivora) from the Late Oligocene of the “Phosphorites du Quercy” (France) and a reappraisal of the genus Cephalogale Geoffroy, 1862. *Geodiversitas* **54**, 787-814 (2013).

78 Bonis, L. d. Contribution à l'Étude des Mammifères de l’Aquitanien de l’Agenais, rongeurs-carnivores-perissodactyles. *Mém. Mus. Natl. d'Hist. Nat.* **28**, 1-192 (1973).

79 Beaumont, G. d. Brèves remarques sur la dentition de certains Ursidés (Mammifères). *Archives des Sciences (Genéve)* **35**, 153-156 (1982).

80 Davis, D. D. The giant panda, a morphological study of evolutionary mechanisms. *Fieldiana Zool. Mem.* **3**, 1-339 (1964).
